# Heterogeneous response of soil organic matter decomposition to temperature change across permafrost regions

**DOI:** 10.1093/nsr/nwag019

**Published:** 2026-01-12

**Authors:** Yan Yang, Shuqi Qin, Tadeo Sáez-Sandino, Leiyi Chen, Siyu Wang, Pete Smith, Luyao Kang, Yuanhe Yang

**Affiliations:** State Key Laboratory of Forage Breeding-by-Design and Utilization, Key Laboratory of Vegetation and Environmental Change, Institute of Botany, Chinese Academy of Sciences, Beijing 100093, China; China National Botanical Garden, Beijing 100093, China; State Key Laboratory of Forage Breeding-by-Design and Utilization, Key Laboratory of Vegetation and Environmental Change, Institute of Botany, Chinese Academy of Sciences, Beijing 100093, China; China National Botanical Garden, Beijing 100093, China; Departamento de Sistemas Físicos, Químicos y Naturales, Universidad Pablo de Olavide, Seville 41013, Spain; State Key Laboratory of Forage Breeding-by-Design and Utilization, Key Laboratory of Vegetation and Environmental Change, Institute of Botany, Chinese Academy of Sciences, Beijing 100093, China; China National Botanical Garden, Beijing 100093, China; State Key Laboratory of Forage Breeding-by-Design and Utilization, Key Laboratory of Vegetation and Environmental Change, Institute of Botany, Chinese Academy of Sciences, Beijing 100093, China; China National Botanical Garden, Beijing 100093, China; College of Resources and Environment, University of Chinese Academy of Sciences, Beijing 100049, China; Institute of Biological and Environmental Sciences, University of Aberdeen, Aberdeen AB24 3UU, UK; State Key Laboratory of Forage Breeding-by-Design and Utilization, Key Laboratory of Vegetation and Environmental Change, Institute of Botany, Chinese Academy of Sciences, Beijing 100093, China; China National Botanical Garden, Beijing 100093, China; State Key Laboratory of Forage Breeding-by-Design and Utilization, Key Laboratory of Vegetation and Environmental Change, Institute of Botany, Chinese Academy of Sciences, Beijing 100093, China; China National Botanical Garden, Beijing 100093, China; College of Resources and Environment, University of Chinese Academy of Sciences, Beijing 100049, China

**Keywords:** temperature sensitivity, soil organic carbon, permafrost, carbon cycle, carbon–climate feedback

## Abstract

Temperature sensitivity (*Q*_10_) of soil organic matter (SOM) releases is a crucial parameter in land surface models for predicting the fate of permafrost carbon (C) under climate warming. Despite its importance, it remains largely unknown whether warming scenarios may cause heterogeneous responses of SOM decomposition in high-latitude and high-altitude permafrost regions. Here, we conduct a 368-day laboratory incubation experiment using topsoil samples collected from 30 sites across high-latitude (Northeast China) and high-altitude (Tibetan Plateau) permafrost regions to elucidate *Q*_10_ patterns and their drivers. We find that the *Q*_10_ value in the high-latitude permafrost regions is ~1.8 times higher than that in the high-altitude permafrost regions. This finding is further supported by a literature synthesis of *Q*_10_ data from the Arctic permafrost regions. Soil microbial properties (that is, microbial composition, α-diversity, life history strategies and C-degrading genes) and mineral protection exert the strongest direct effects on *Q*_10_ variation, whereas climate (aridity), soil characteristics (pH and clay + silt) and substrate quantity influence *Q*_10_ indirectly across the two permafrost regions. These findings highlight that permafrost regions at high latitudes, characterized by higher *Q*_10_ values and substrate quantity, may be more susceptible to C losses under warming scenarios compared with high-altitude permafrost regions.

## INTRODUCTION

Permafrost, characterized by soils or rocks that remain below 0°C for at least two consecutive years, is mainly located in high-latitude and high-altitude regions across the Northern Hemisphere [1], covering approximately 15% of the land surface [2]. Permafrost regions store approximately 1014–1035 Pg (1 Pg = 10^15^ g) of organic carbon (C) in the top 3-m soils, which is nearly double the atmospheric C pool and represents one-third of the global soil C storage [3,4]. During the recent two decades, temperatures in these regions have been increasing two to four times faster in the rest of the world [5–7]. Warming-induced permafrost thaw may expose a substantial reservoir of previously frozen organic C accessible to microbial decomposition and result in substantial soil C emissions [8,9], which could then trigger a positive permafrost C–climate feedback [10,11]. However, the predicted soil C release upon permafrost thaw exhibits substantial uncertainties among land surface models [12,13]. One of the primary sources of model uncertainty is that land surface models assume a constant value for temperature sensitivity of soil organic matter (SOM) decomposition (*Q*_10_; the factor that quantifies how the decomposition rate changes in response to a 10°C temperature increase) [14,15]. To improve model parameterization, it is crucial to explore the spatial variability and key drivers of *Q*_10_, which will help reduce uncertainties in model projections of soil C emissions across permafrost regions even around the world.

Despite a long-standing interest in this topic [16,17], our understanding on patterns and drivers of *Q*_10_ in permafrost regions remains inadequate [17]. Up to now, prior studies have mainly focused on either high-latitude [18,19] or high-altitude [16,20] permafrost regions; no studies have assessed *Q*_10_ patterns in these two regions using standardized protocols at the same time. Identifying the potential differences of soil C losses to climate warming between different permafrost regions is highly essential for providing valuable insights that can aid in developing climate change mitigation strategies [21]. More importantly, it remains unclear whether and how multiple potential drivers (environmental factors, biochemical recalcitrance and soil microbiome) jointly explain the variations in *Q*_10_ across different permafrost regions [22]. In particular, microbial properties have not been incorporated into the predictions of *Q*_10_ patterns across global permafrost regions [23]. These knowledge gaps cause large uncertainties in land surface models when predicting the direction and magnitude of permafrost C–climate feedback in a warmer world.

Here, we evaluated spatial variations and dominant drivers of *Q*_10_ across the southern edge of a high-latitude permafrost region (Northeast China) and a high-altitude permafrost region (the Tibetan Plateau) (see Fig. 1a–c for geographic locations). To this end, we conducted a large-scale laboratory incubation by collecting topsoil samples from 30 study sites (Table S1), and also synthesized *Q*_10_ observations from Arctic permafrost regions to capture broader spatial patterns. To identify key factors mediating *Q*_10_ [17], we further examined environmental factors (climate and soil properties), substrate properties [i.e. soil organic carbon (SOC) and alkyl/O-alkyl ratio], mineral protection [i.e. the concentration of SOC associated with cation bridges (OC–Ca)] and soil microbiome (i.e. microbial community composition and C-degrading genes). Based on these observations and measurements, we aimed to test the hypothesis that high-latitude permafrost regions could exhibit higher *Q*_10_ values than high-altitude permafrost areas because of the following considerations. Specifically, high-latitude areas, dominated by forests and shrubs, which have greater primary productivity than grassland-dominated high-altitude permafrost regions [20], accumulate more SOC [24] and receive substantial inputs of recalcitrant compounds (low-quality substrates) from forests and shrubs [25]. The greater SOC content [26] and the more recalcitrant compounds, which require higher activation energy (carbon-quality temperature hypothesis) [19,27], together with the cooperative depolymerization by a consortium of soil microorganisms, could jointly influence *Q*_10_ [27]. Moreover, when soil pH is >6, Ca^2+^ is more prevalent, potentially enhancing SOC stabilization through Ca^2+^ bridging (inner- and outer-sphere complexation) or Ca-mediated aggregation [28,29]. Consequently, the alkaline soils in high-altitude permafrost regions may promote stronger SOC stabilization than the acidic soils across high-latitude permafrost areas. Together, these contrasting mechanisms might result in higher *Q*_10_ values in high-latitude than in high-altitude permafrost regions.

**Figure 1.**
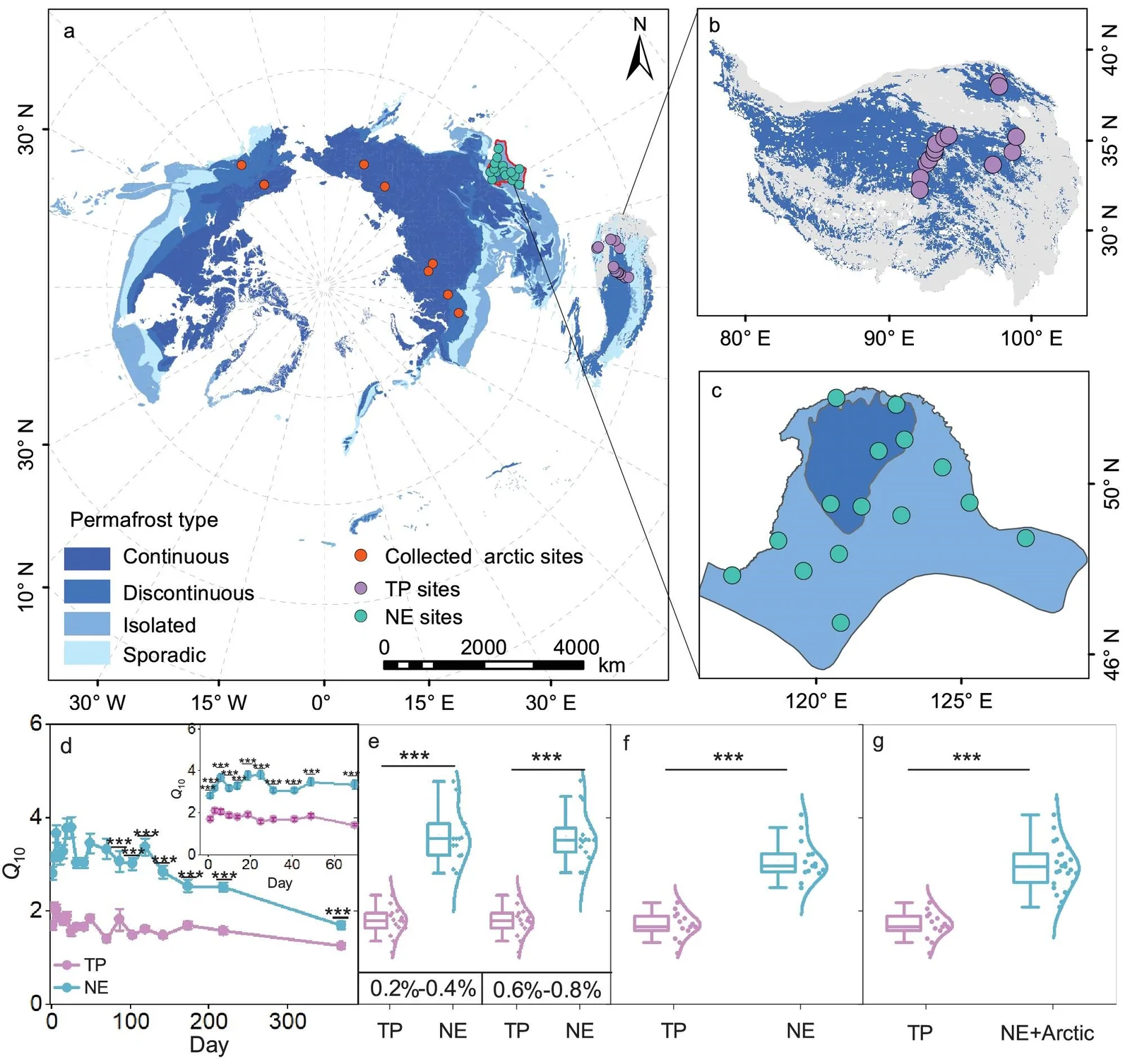
Sampling sites and *Q*_10_ of soil organic carbon decomposition *Q*_10_ across high-altitude and high-latitude permafrost regions. (a) Data distribution of Arctic permafrost and sampling sites based on Circum-Arctic map of permafrost. (b) Distribution of sampling sites in high-altitude permafrost region on the Tibetan Plateau and (c) high-latitude permafrost region of Northeast China. (d) *Q*_10_ results from 18 measurements across these two regions. The inset in panel (d) displays *Q*_10_ results from the first 70 days of incubation. (e) Comparison of *Q*_10-q_ values at cumulative respired soil C in the ranges of 0.2%–0.4% and 0.6%–0.8% between the high-altitude permafrost region on the Tibetan Plateau and the high-latitude permafrost region of Northeast China. (f) Comparison of *Q*_10_ values between the high-altitude permafrost region on the Tibetan Plateau and the high-latitude permafrost region of Northeast China. (g) *Q*_10_ comparison between the high-altitude permafrost region on the Tibetan Plateau and the high-latitude permafrost regions of Northeast China and the Arctic. *Q*_10_ values were calculated using the ratio of soil CO_2_ release rates at warmer and colder temperatures over the same incubation period, whereas *Q*_10-q_ values were determined by dividing the time taken to respire a given fraction of SOC at 5°C by that at 15°C. The line graph shows average values ± standard error. Boxes represent the interquartile range (25th to 75th percentiles), with horizontal lines within the boxes indicating the median values, and dots indicating the mean values. The whiskers extend to 1.5 times the interquartile range. Data distributions are displayed on the right side of boxes, with TP (*n* = 15), NE (*n* = 15) and Arctic (*n* = 10) representing the permafrost regions of the Tibetan Plateau, Northeast China and the Arctic, respectively. The Wilcoxon rank-sum test was used to determine the significant differences of *Q*_10_ values across two regions. *** *P* < 0.001. The permafrost distribution map for the Northern Hemisphere was obtained from the National Snow and Ice Data Center [68]. The permafrost distribution is classified as follows: continuous permafrost (covering 90%–100% of the landscape), discontinuous permafrost (50%–90%), sporadic permafrost (10%–50%) and isolated patches (0.5%–10%) [69]. The permafrost map on the Tibetan Plateau was sourced from Zou *et al*. [70]. Review drawing number: GS 京 (2026) 2449号.

## RESULTS AND DISCUSSION

### Higher *Q*_10_ in high-latitude than in high-altitude permafrost regions

Consistent with the hypothesis mentioned above, our results revealed that *Q*_10_ values were significantly higher in Northeast China (high-latitude permafrost region) than on the Tibetan Plateau (high-altitude permafrost region) throughout the entire incubation period, with average values of 3.1 ± 0.1 and 1.7 ± 0.1, respectively (data are presented as mean ± standard errors unless noted otherwise; *P* < 0.001, Fig. 1f). To validate the results obtained from the ‘equal-time’ method [30], we calculated soil *Q*_10-q_ values based on the ‘equal-C’ approach [31], in which *Q*_10-q_ values were determined at cumulative respiratory soil C levels of 0.2%–0.4% and 0.6%–0.8% (see details in the Methods section of the Supplementary Data). The results showed that *Q*_10-q_ values were positively correlated with *Q*_10_ (all *P* < 0.001; Fig. S1), and exhibited similar patterns to those obtained using the ‘equal-time’ method (Fig. 1e). We then adopted *Q*_10_ measurements derived from the ‘equal-time’ method [30,32] for subsequent analyses to facilitate comparison with earlier studies conducted in the Arctic region. The synthesis of *Q*_10_ data from nine individual sites in Arctic permafrost regions (Reference list S1) confirmed that *Q*_10_ values from the high-latitude permafrost regions were significantly higher than those from the high-altitude permafrost regions (*P* < 0.001; Fig. 1g). Additionally, our results showed that *Q*_10_ values in isolated permafrost regions were significantly higher than in discontinuous and sporadic permafrost areas (*P* < 0.05; Fig. S2), possibly due to the larger vegetation productivity (*P* < 0.05; Fig. S3). The stronger vegetation growth, dominated by conifers and shrubs, likely leads to increased inputs of recalcitrant litter [33], which in turn contributes to the higher *Q*_10_ values (see Note S1 for detailed discussion).

The higher *Q*_10_ observed in high-latitude than in high-altitude permafrost regions could be attributed to various abiotic and biotic factors. To illustrate this point, we detected significant differences in most variables between the two permafrost regions (Fig. 2; see Notes S2 and S3 for details), and explored their relationships with *Q*_10_. Based on the variables that showed differences between the two permafrost regions (Figs 2 and 3) and their associations with *Q*_10_ (Fig. 4), we then employed structural equation modelling (SEM) to elucidate direct and indirect drivers of *Q*_10_. Before conducting SEM, principal component analyses were performed for each group of variables (Table S2), followed by variance inflation factors tests (Table S3), which revealed the limited multicollinearity among each group of variables. The subsequent SEM analysis pinpointed that soil microbiome (microbial community composition, α-diversity, life history strategies and C-degrading genes) and mineral protection (the proportion of OC–Ca) exerted the strongest direct positive and negative associations with *Q*_10_ values, respectively (Fig. 5). Considering that substrates and microbial communities might change during the 368-day incubation, we further examined their potential effects on the observed patterns and drivers of *Q*_10_ by conducting additional SEM analyses for *Q*_10_ values derived from the short-term incubation (3 weeks; Fig. S4), as well as for *Q*_10-q_ values calculated on the basis of the ‘equal-C’ method (Fig. S5). These supplementary analyses confirmed the main findings derived from the long-term incubation, highlighting that the potential changes in substrate and microbial properties over the incubation period were insufficient to alter patterns and drivers of *Q*_10_ observed in this study.

**Figure 2.**
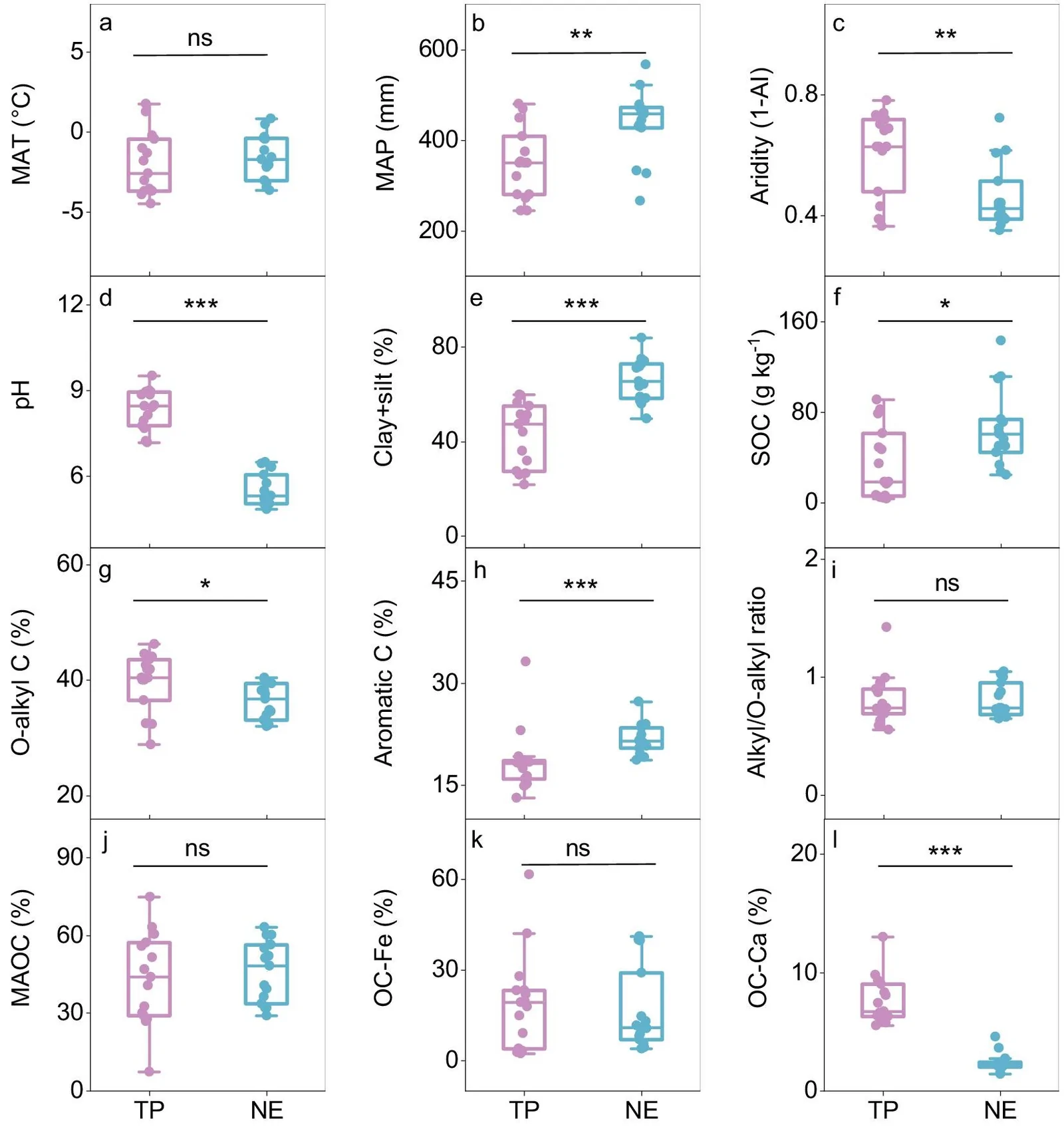
Comparison of climate, soil properties, substrate properties and mineral protection in the high-altitude permafrost region on the Tibetan Plateau (TP) with the high-latitude permafrost region of Northeast China (NE). (a–i) Values for: MAT (a); mean annual precipitation (MAP) (b); aridity (1−aridity index, with higher values indicating greater aridity (c); pH (d); clay + silt content (e); SOC (f); O-alkyl C (g); Aromatic C (h); and Alkyl/O-alkyl C ratio (i). (j–l) The proportion of C associated with: MAOC (j); OC–Fe (k), and OC–Ca (l). Boxes represent the interquartile range (25th to 75th percentiles), with horizontal lines within the boxes indicating the median values. The whiskers extend to 1.5 times the interquartile range. The Wilcoxon rank-sum test was used to assess significant differences between TP and NE. * *P* < 0.05, ** *P* < 0.01 and *** *P* < 0.001, while ‘ns’ denotes no significant difference.

**Figure 3.**
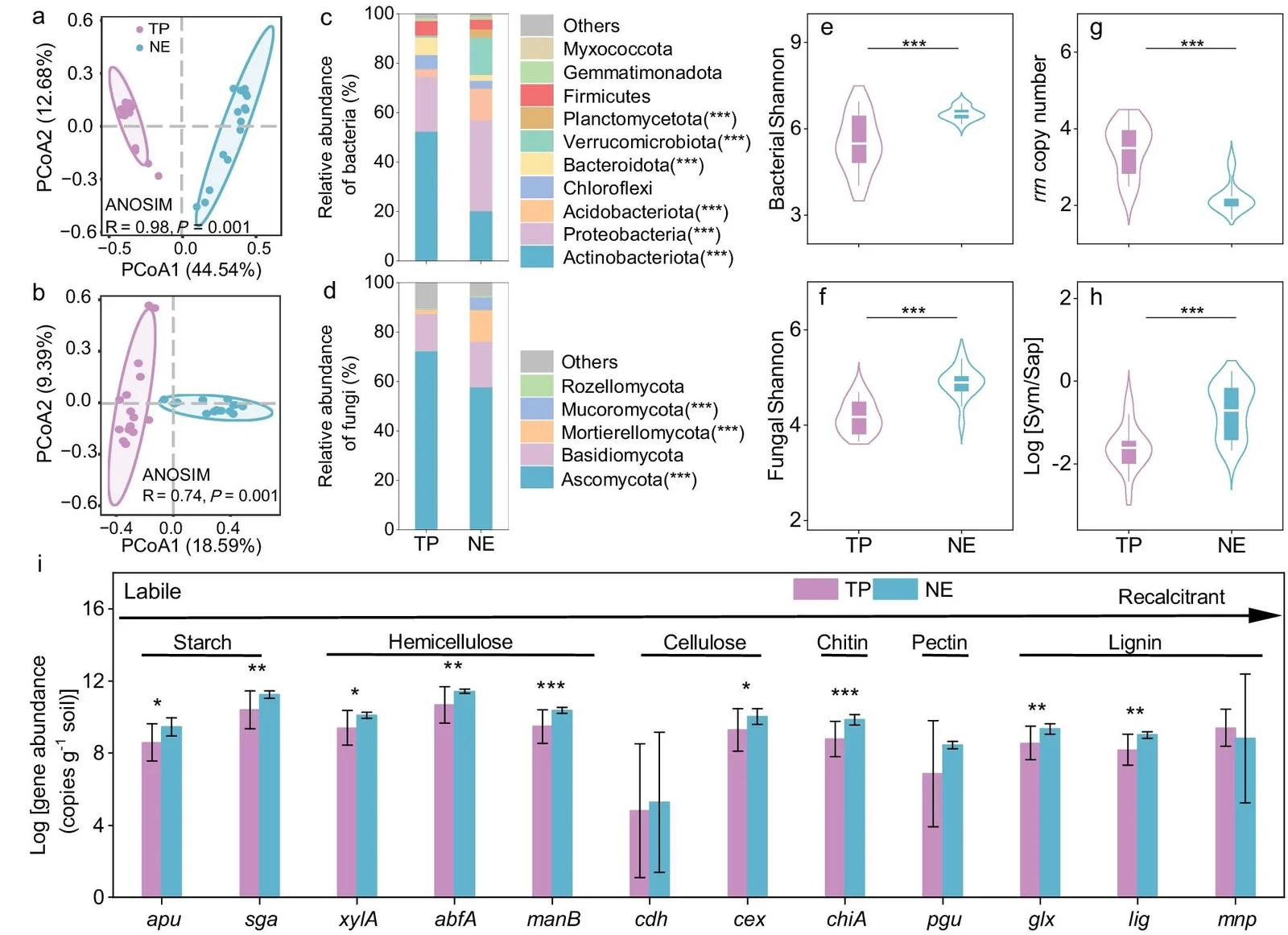
Comparison of microbial community composition, α-diversity, life history strategies and C-degrading genes in the high-altitude permafrost region on the Tibetan Plateau (TP) with the high-latitude permafrost region of Northeast China (NE). Principal coordinate analysis (PCoA) of bacterial (a) and fungal communities (b) in the two permafrost regions based on the Bray–Curtis dissimilarities. Ellipses denote 95% confidence intervals. Stacked bar charts show the relative abundances of bacterial (c) and fungal phyla (d). Violin plots of Shannon index for bacteria (e) and fungi (f), community-weighted *rrn* copy numbers for bacteria (g), and the ratio of Sym/Sap fungi (log-transformed) (h). Bar chart shows the abundances of C-degrading genes (i). The degrading genes included those associated with the degradation of starch (*apu* and *sga*), hemicellulose (*xylA, abfA* and *manB*), cellulose (*cdh* and *cex*), chitin (*chiA*), pectin (*pgu*) and lignin (*glx, lig* and *mnp*) (Table S5). Boxes within the violin plots represent the interquartile range (25th to 75th percentiles), with horizontal lines depicting median values, and the whiskers extending to 1.5 times the interquartile range. Error bars in the bar charts represent standard errors (*n* = 15). Microbial community dissimilarities between the two permafrost regions were assessed by analysis of similarities (ANOSIM). The Wilcoxon rank-sum test was used to assess significant differences between TP and NE. * *P* < 0.05, ** *P* < 0.01 and *** *P* < 0.001.

**Figure 4.**
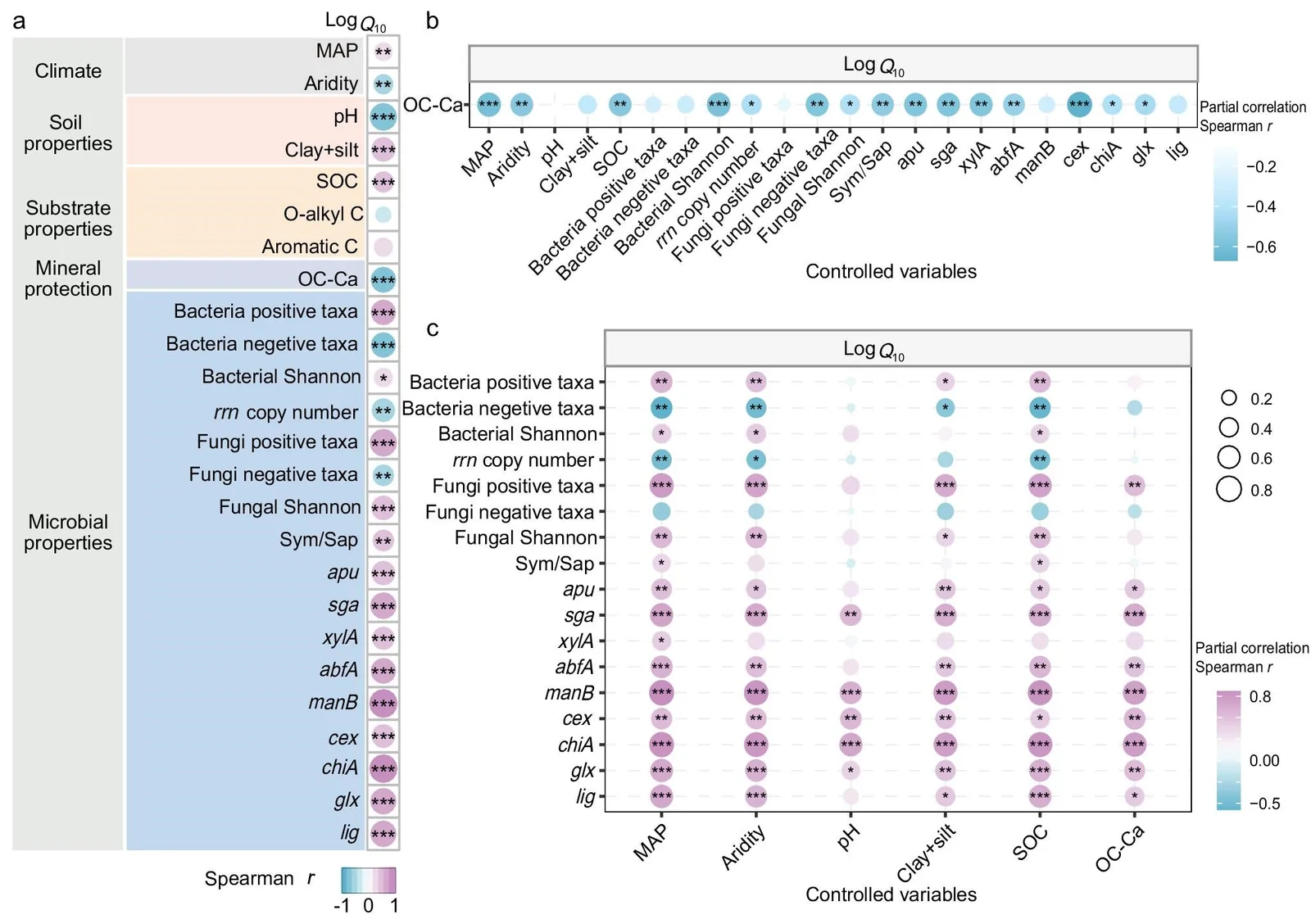
Correlations of *Q*_10_ of soil organic carbon decomposition with abiotic and biotic variables. (a) Spearman correlations between *Q*_10_ of soil organic carbon decomposition and five categories of predictors (climate, soil properties, substrate properties, mineral protection and microbial properties). Partial correlations between *Q*_10_ of soil organic carbon decomposition and mineral protection (the proportion of OC–Ca) (b), as well as microbial properties (c). In the partial correlation, the *x*-axis shows the controlled variables, and the *y*-axis displays mineral protection (OC–Ca) and microbial properties related to *Q*_10._ values MAT, OC–Fe and MAOC were excluded from the Spearman correlation analysis due to their lack of significant differentiation between the two regions. Climate variables include mean annual precipitation (MAP) and aridity. Soil properties consist of pH and clay + silt. Substrate properties comprise SOC, O-alkyl C and aromatic C. Mineral protection is indicated by OC–Ca. Microbial compositions were represented by aggregating dominant phyla positively/negatively correlated with *Q*_10_. Bacterial positive/negative taxa were identified by aggregating dominant phyla that were positively/negatively correlated with *Q*_10_ following the method described by Sáez-Sandino *et al*. [17]. Fungal positive/negative taxa were defined using a method analogous to that employed for bacteria. The microbial attributes also included the Shannon index of bacteria and fungi, community-weighted *rrn* copy number, the ratio of Sym/Sap fungi, and the abundance of C-degrading functional genes (*apu, sga, xylA, abfA, manB, cex, chiA, glx* and *lig*). The size and color of the circles correspond to the intensity and direction of the correlations, respectively. * *P* < 0.05, ** *P* < 0.01 and *** *P* < 0.001.

**Figure 5.**
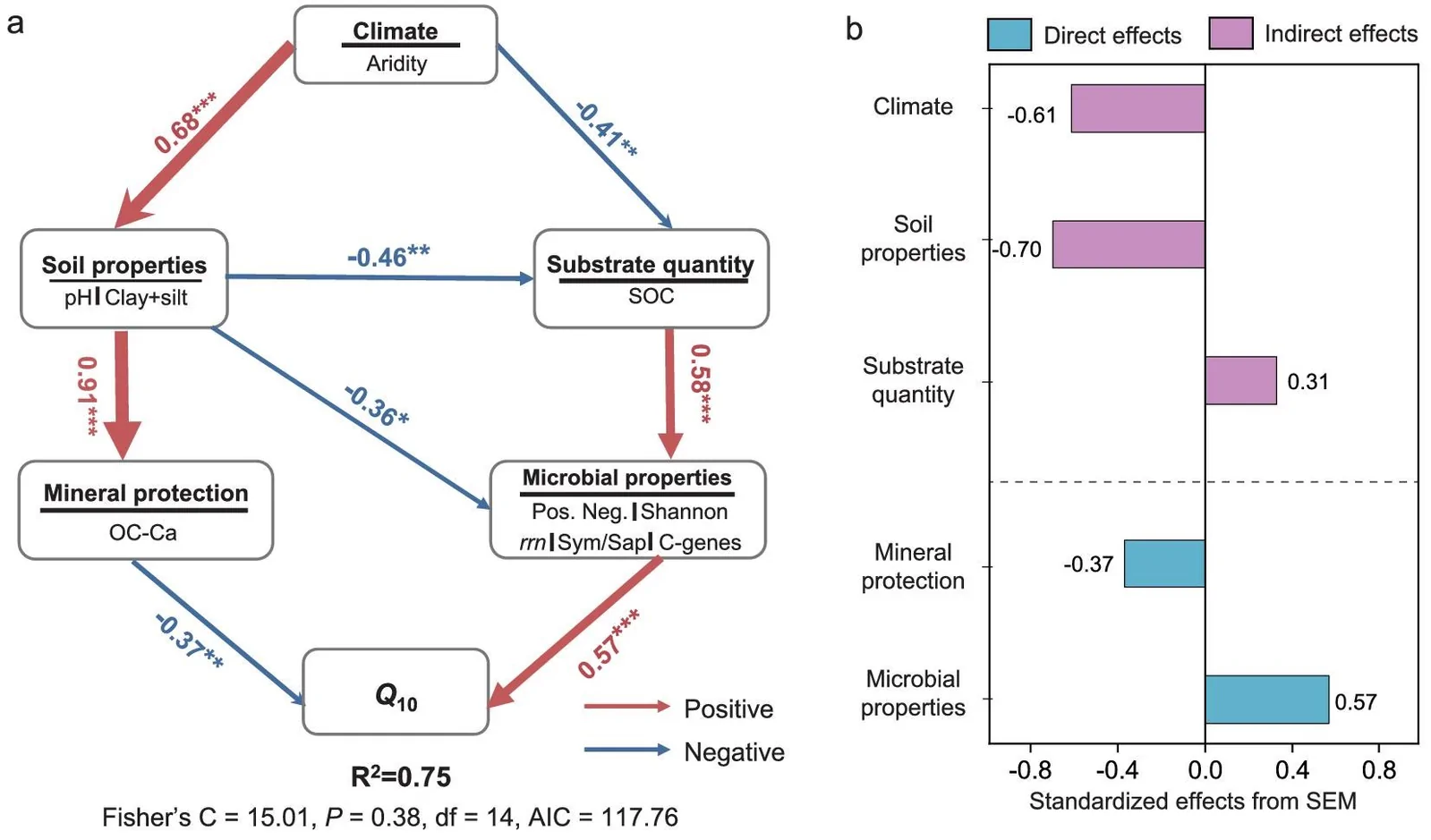
Ecological predictors of *Q*_10_ of soil organic carbon decomposition variation. (a) The SEM depicts direct and indirect effects of climate, soil properties, substrate quantity, mineral protection and microbial properties on *Q*_10_ variation. (b) Standardized direct effects of mineral protection and microbial properties, as well as indirect effects of climate and soil properties. Single arrows denote hypothesized causation. Red and blue arrows indicate significant positive and negative pathways, respectively. The numbers adjacent to the arrow represent the magnitude of the standardized path coefficients, which correspond to the width of the arrows. Climate is represented by aridity (1 − aridity index), with higher values indicating greater aridity. Soil and microbial properties are captured by the extracted PC1 axis. Soil properties include pH and clay + silt content. Substrate quantity comprises SOC. Mineral protection is indicated by the proportion of OC–Ca. Microbial properties involve the bacterial positive/negative taxa, fungal positive/negative taxa, Shannon index of bacteria and fungi, community-weighted *rrn* copy number, the ratio of Sym/Sap fungi and the abundances of C-degrading functional genes (*apu, sga, xylA, abfA, manB, cex, chiA, glx* and *lig*). Bacterial positive/negative taxa were identified by aggregating dominant phyla that were positively/negatively correlated with *Q*_10_ (following the method described by Sáez-Sandino *et al.* [17]). Fungal positive/negative taxa were defined using a method analogous to that employed for bacteria. * *P* < 0.05, ** *P* < 0.01 and *** *P* < 0.001.

### Microbial properties are associated with *Q*_10_ variations between high-latitude and high-altitude permafrost regions

The SEM results revealed that microbial properties (microbial community composition, α-diversity, life history strategies and C-degrading genes) had a significant direct effect on *Q*_10_ (Fig. 5). At the phylum level, dominant microbial phyla were positively or negatively correlated with *Q*_10_ values (Fig. S6). Specifically, Proteobacteria and acidophilic Acidobacteria, characterized by broad substrate utilization capacity and the ability to degrade recalcitrant compounds such as chitin [34,35], were positively associated with *Q*_10_ (*P* < 0.001 and *P* < 0.05, respectively; Fig. S6). In contrast, Actinobacteria, which share similar ecological niches with Acidobacteria and experience stronger competitive pressure under acidic conditions [36], were negatively correlated with *Q*_10_ (*P* < 0.001; Fig. S6). In other words, the positive correlations between microbial taxa and *Q*_10_ may result from a broader enzymatic capacity that enhances decomposition under warming, whereas the negative correlations likely reflect competitive constraints that limit microbial activity in certain taxa [34,36]. At the amplicon sequence variants (ASVs) level, 1519 ASVs of soil bacteria and fungi were positively or negatively associated with *Q*_10_, with the number of positively correlated ASVs being nearly twice that of negatively correlated ones (Table S4). Moreover, their relative abundance represented 26.7% of the bacterial and 27.0% of the fungal communities, suggesting that soil *Q*_10_ might be primarily mediated by a limited subset of microorganisms despite soil C mineralization being a generalist function carried out by most microbial groups [17,37].

Our results also illustrated the positive correlation between *Q*_10_ values and microbial diversity (Fig. 4), possibly due to the strong microbial interactions within highly diverse communities. According to the biodiversity complementary theory [38], multiple species can functionally complement one another to maintain the homeostasis of SOM decomposition under a changing environment [38]. Microbial communities with higher α-diversity are likely to experience more frequent interspecific interactions (e.g. mutualism, competition and antagonism) [39], which may further enhance the microbial-mediated C release and its response to temperature changes [39]. To test this possibility, we established bacterial–fungal co-occurrence networks for both permafrost regions (see Note S4 for details). Our results revealed that the microbial co-occurrence network in the high-latitude permafrost regions was more complex, with a larger network size (total nodes) and higher connectivity (total links) compared to the high-altitude permafrost regions (Fig. S7). This increased network complexity [40,41], together with a higher proportion of positive associations between species in high-latitude permafrost regions (Northern China vs. Tibetan Plateau: 71.3% vs. 60.6%) indicated extensive cooperative behaviors among microbial taxa [40,42]. Such cooperative relationships would then amplify the soil *Q*_10_ due to the fact that fungi can produce intermediate compounds during the decomposition of recalcitrant substrates which, in turn, stimulate bacterial growth [37,43].

Our results further demonstrated that *Q*_10_ patterns were related to microbial K/r strategies (Fig. 4). Specifically, higher *Q*_10_ values were detected in regions enriched in K-strategists, revealed by the lower bacterial community weighted ribosomal RNA (*rrn*) copy numbers and a greater proportion of oligotrophic fungi [symbiotrophic to saprotrophic (Sym/Sap) fungi] (all *P* < 0.01; Fig. 4a). This situation may be attributed to K/r strategists decomposing SOM with different activation energies [44,45]. It has been reported that K-strategists are capable of decomposing lignin and aromatic phenolic compounds, both of which possess high activation energies [44], while r-strategists could prefer to utilize labile SOM with low activation energy [44,46]. Consistent with this deduction, our results revealed that bacterial *rrn* copy numbers were negatively associated with aromatic C content (*P* < 0.01; Fig. S8b), but positively linked to O-alkyl C content (*P* < 0.001; Fig. S8a). Likewise, the Sym/Sap (K/r) ratios in fungal communities were positively correlated with aromatic compounds (*P* < 0.05; Fig. S8d). Correspondingly, high-latitude permafrost regions exhibited lower *rrn* copy numbers (*P* < 0.001; Fig. 3g) and higher Sym/Sap ratios (*P* < 0.001; Fig. 3h) than high-altitude permafrost regions, together with more aromatic compounds (*P* < 0.001; Fig. 2h) leading to higher *Q*_10_ values. Aside from microbial life history strategies, our results showed that C degradation genes were positively associated with soil *Q*_10_ (Fig. 4). It has been reported that the high expression of certain genes reflects an enhanced potential for the decomposition of corresponding substrates [47]. In our case, the abundances of the lignin-degrading gene (*lig*, encoding lignin peroxidase) and aromatic/terpene-degrading genes (*glx*, encoding glycolate oxidase) were higher in high-latitude than in high-altitude permafrost regions (all *P* < 0.01; Fig. 3i), reflecting a greater potential for the degradation of chemically complex substrates [48]. Consequently, higher abundances of C degradation genes, particularly those involved in the breakdown of recalcitrant C, may enhance the responsiveness of soil C mineralization to temperature changes in high-latitude permafrost regions [49].

### Linkage of mineral protection with *Q*_10_ differences between high-latitude and high-altitude permafrost regions

The SEM results indicated that mineral protection (the proportion of OC–Ca) exerted a significant direct negative effect on *Q*_10_ (Fig. 5). The negative association between the proportion of OC–Ca and *Q*_10_ values (*P* < 0.001; Fig. 4) was supported by a previous study, which highlighted the role of Ca^2+^ in mediating C release and temperature sensitivity in permafrost soils on the Tibetan Plateau [16]. Such a pattern could be explained by the mineral protection mechanism that directly reduces the bioavailability of organic C [29]. It is well known that minerals can limit the accessibility of microorganisms and enzymes to C substrates through physicochemical mechanisms such as cation bridging [29] and ligand exchange [50], thus diminishing the *Q*_10_ of SOM decomposition. In alkaline and calcareous soils, polyvalent cations like Ca^2+^ are prevalent [16]. The Ca^2+^ protects SOM from biodegradation by forming bridges with negatively charged phyllosilicates and SOM [16,29]. In our case, the proportion of OC–Ca was significantly higher on the Tibetan Plateau than that in Northern China (*P* < 0.001; Fig. 2l), which could be due to the fact that high-altitude permafrost regions are dominated by alkaline soils, whereas high-latitude permafrost areas are typically characterized with acidic soils (Fig. 2d). Consequently, the higher OC–Ca content on the Tibetan Plateau may reduce substrate availability, which in turn leads to a lower *Q*_10_.

Besides Ca^2+^, Fe-(hydr) oxides, which are characterized by abundant hydroxyl groups, can engage in ligand exchange with hydroxyl or phenolic groups in SOM, thereby constraining SOM availability [50,51]. Additionally, organic C bound to the mineral surface forms mineral-associated organic matter (MAOM), which restricts C substrate diffusion, microbial decomposition and their response to temperature changes [52,53]. However, in contrast to these theoretical considerations, our results revealed that neither the proportion of mineral-associated organic carbon (MAOC) (*P* = 0.6; Fig. 2j) nor the proportion of iron-bound organic carbon (OC–Fe) (*P* = 0.3; Fig. 2k) showed differences between the two permafrost regions, suggesting that these mechanisms do not contribute to the observed regional variations in *Q*_10_. This finding also contrasted with those derived from Arctic permafrost soils, where MAOC and OC–Fe content have been shown to suppress *Q*_10_ [19]. The discrepancy might arise because mean annual temperature (MAT), a key predictor of global MAOC and OC–Fe distribution [54,55], did not differ significantly between Northern China and the Tibetan Plateau (*P* = 0.3; Fig. 2a), potentially resulting in similar MAOC and OC–Fe levels and thus non-significant effects of MAOC and Fe oxides on *Q*_10_.

### Effects of climatic, edaphic and substrate properties on *Q*_10_ variations across high-latitude and high-altitude permafrost regions

Aridity played a significant role in the indirect mediation of *Q*_10_ variations across high-latitude and high-altitude permafrost regions. First, as shown by the SEM results (Fig. 5), with increasing aridity, lower SOC storage led to a decrease in microbial diversity, ultimately suppressing *Q*_10_. Such a pathway likely reflected the situation that the intensified water limitation, which not only constrained below-ground C inputs but also acted as an environmental filter, thereby reducing microbial diversity [56]. The decline in microbial diversity might have led to a loss of species capable of enhancing metabolic activity in response to temperature increases [57], resulting in a lower *Q*_10_. Second, as illustrated by the SEM results (Fig. 5), aridity could suppress *Q*_10_ by increasing OC–Ca content, possibly through reduced substrate accessibility. It has been shown that aridity-induced soil salinization increased the opportunities for inner- and outer-sphere interactions between organic C and cations [22]. In support of this argument, our results also demonstrated the increasing OC–Ca content along aridity gradients (*P* < 0.01; Fig. S9). Consequently, high-altitude permafrost regions characterized by greater aridity could enhance Ca^2+^ protection, leading to reduced substrate availability and consequently resulting in lower *Q*_10_. In addition, aridity would limit plant growth and promote conservative strategies characterized by higher root tissue density and lower root exudation [58], which might constrain soil substrate availability and consequently reduce the apparent *Q*_10_. Nevertheless, future studies should quantify these functional traits to clarify their role in shaping soil *Q*_10_ variations across permafrost regions.

Among edaphic properties, soil pH played a pivotal role in mediating *Q*_10_ (Fig. S10a) through two coupled pathways: influencing microbial life history strategies; and mediating mineral–organic associations (Fig. 5). On the one hand, under acidic conditions, microbial communities tended to shift toward oligotrophic strategies [59]. In line with this point, our results showed that lower pH values corresponded with reduced *rrn* copy numbers (*P* < 0.001; Fig. S10b) and higher Sym/Sap ratio (*P* < 0.001; Fig. S10c), reflecting the predominance of oligotrophic microorganisms in acidic soils. These oligotrophic microorganisms are typically enriched in function related to resource scavenging, such as the degradation of complex organic compounds [59]. Consequently, the dominance of oligotrophic microorganisms in acidic, high-latitude permafrost ecosystems may enhance the decomposition of complex substrates, thereby increasing *Q*_10_. On the other hand, the SEM results showed that increasing soil pH indirectly suppressed *Q*_10_ by increasing OC–Ca content (Fig. 5), likely due to enhanced mineral stabilization of organic matter. It has been reported that as pH increases, the adsorption of organic compounds by Ca^2+^ strengthens [60], consistent with the observed positive correlation between pH and OC–Ca content (*P* < 0.001; Fig. S10d). Based on these facts, we infer that in high-altitude permafrost regions, high pH levels enhance the protective effect of Ca^2+^ on organic matter, thereby limiting substrate availability and ultimately reducing *Q*_10_.

SOM quality (O-alkyl C and aromatic C) has been widely recognized as a key factor in mediating *Q*_10_ [52], but our study did not detect a direct mediating effect of substrate quality on *Q*_10_ values (Fig. 4), highlighting that, compared to substrate quality, *Q*_10_ variations across contrasting permafrost regions are more dependent on other abiotic and biotic factors (such as microbial properties and mineral protection, as observed in our study). In contrast to substrate quality, the SEM results revealed that SOC content served as a substrate source for microbes, which modulated *Q*_10_ through soil microbial properties (Fig. 5). Typically, higher SOC content supports greater microbial diversity and genes involved in SOM decomposition [61,62], as also supported by our SEM results (Fig. 5). Once low temperature constraints are alleviated in permafrost regions, a larger proportion of the microbial community becomes metabolically active [61], potentially enhancing microbial respiration and increasing *Q*_10_.

## CONCLUSION

Based on the combination of broad geographical soil sampling, long-term laboratory incubation and integration of observational dataset from Arctic permafrost regions, our study provided the experimental evidence that *Q*_10_ values in permafrost regions are higher in high-latitude than in high-altitude permafrost regions. These observations established a crucial benchmark for assessing model simulations of permafrost C–climate feedback. Our findings indicated the divergent response of soil microbial respiration to changing temperature across different permafrost regions. Given that Northeast China with higher SOC content has also experienced both greater historical warming and stronger projected warming under the SSP5–8.5 scenario for 2080–99 period than the Tibetan Plateau [63], high-latitude permafrost regions may be more sensitive and act as hotspots for CO_2_ emissions under global warming scenarios. Furthermore, our results demonstrated that microbial properties (microbial community composition, α-diversity, life history strategies and functional genes) and mineral protection exerted dominant direct effects on *Q*_10_ variations compared to other factors. This finding underscores the necessity of incorporating microbial and mineral properties into land surface models. Although some models integrate microbial biomass as a quantitative proxy and include certain functional genes [64–66], these models often overlook other critical microbial metrics, such as microbial community composition and life history strategies. Additionally, current land surface models frequently neglect soil mineral interactions, despite mineral protection being known to stabilize SOC and inhibit microbial decomposition [19,67]. Overall, future model improvements should prioritize the above mechanisms to reduce uncertainty in predicting permafrost C dynamics under climate warming.

## METHODS

This study was mainly conducted on the Tibetan Plateau and in Northeast China, which represent high-altitude and high-latitude permafrost regions, respectively (Fig. 1a–c). Across the two studied permafrost regions, we established 15 representative sites on the Tibetan Plateau and 15 in Northeast China, and conducted long-term laboratory incubation of surface soils to assess spatial variations in soil *Q*_10_ between high-altitude and high-latitude permafrost regions. In this 368-day incubation experiment, we measured CO_2_ release rates and calculated *Q*_10_ using both ‘equal-time’ and ‘equal-C’ methods [30,31]. To evaluate the generalizability of the patterns observed across the two regions, we synthesized *Q*_10_ measurements from Arctic permafrost regions obtained under the same incubation conditions. We further quantified a suite of biotic and abiotic attributes, including climate, soil physicochemical properties, substrate quantity and quality, mineral protection and soil microbial properties. Finally, we used Spearman correlations and SEM to disentangle the key drivers of regional variation in *Q*_10_ between high-latitude and high-altitude permafrost regions. Detailed descriptions of the methods are available in the online Supplementary Data.

## Supplementary Material

nwag019_Supplemental_Files

## References

[1] Zhang T, Barry RG, Knowles K et al. Statistics and characteristics of permafrost and ground-ice distribution in the Northern Hemisphere. Polar Geogr 1999; 23: 132–54. 10.1080/10889379909377670

[2] Obu J . How much of the Earth’s surface is underlain by permafrost? J Geophys Res Earth Surf 2021; 126: e2021JF006123. 10.1029/2021JF006123

[3] Hugelius G, Strauss J, Zubrzycki S et al. Estimated stocks of circumpolar permafrost carbon with quantified uncertainty ranges and identified data gaps. Biogeosciences 2014; 11: 6573–93. 10.5194/bg-11-6573-2014

[4] Mishra U, Hugelius G, Shelef E et al. Spatial heterogeneity and environmental predictors of permafrost region soil organic carbon stocks. Sci Adv 2021; 7: eaaz5236. 10.1126/sciadv.aaz5236 PMC7904252 33627437

[5] Pepin N, Bradley RS, Diaz HF et al. Elevation-dependent warming in mountain regions of the world. Nat Clim Change 2015; 5: 424–30. 10.1038/nclimate2563

[6] Biskaborn BK, Smith SL, Noetzli J et al. Permafrost is warming at a global scale. Nat Commun 2019; 10: 264. 10.1038/s41467-018-08240-4 PMC6335433 30651568

[7] Bai Y, Peng Y, Zhang D et al. Heating up the roof of the world: tracing the impacts of *in-situ* warming on carbon cycle in alpine grasslands on the Tibetan Plateau. Natl Sci Rev 2025; 12: nwae371. 10.1093/nsr/nwae371 PMC11771398 39872222

[8] Koven CD, Ringeval B, Friedlingstein P et al. Permafrost carbon-climate feedbacks accelerate global warming. Proc Natl Acad Sci USA 2011; 108: 14769–74. 10.1073/pnas.1103910108 PMC3169129 21852573

[9] Osterkamp T, Jorgenson M, Schuur E et al. Physical and ecological changes associated with warming permafrost and thermokarst in interior Alaska. Permafrost Periglac 2009; 20: 235–56. 10.1002/ppp.656

[10] Walter Anthony K, Schneider von Deimling T, Nitze I et al. 21st-century modeled permafrost carbon emissions accelerated by abrupt thaw beneath lakes. Nat Commun 2018; 9: 3262. 10.1038/s41467-018-05738-9 PMC6093858 30111815

[11] Chen L, Yang G, Bai Y et al. Permafrost carbon cycle and its dynamics on the Tibetan Plateau. Sci China Life Sci 2024; 67: 1833–48. 10.1007/s11427-023-2601-1 38951429

[12] Todd-Brown K, Randerson J, Hopkins F et al. Changes in soil organic carbon storage predicted by Earth system models during the 21st century. Biogeosciences 2014; 11: 2341–56. 10.5194/bg-11-2341-2014

[13] McGuire AD, Lawrence DM, Koven C et al. Dependence of the evolution of carbon dynamics in the northern permafrost region on the trajectory of climate change. Proc Natl Acad Sci USA 2018; 115: 3882–7. 10.1073/pnas.1719903115 PMC5899459 29581283

[14] Friedlingstein P, Cox P, Betts R et al. Climate–carbon cycle feedback analysis: results from the C^4^MIP model intercomparison. J Clim 2006; 19: 3337–53. 10.1175/JCLI3800.1

[15] Sun W, Luo X, Fang Y et al. Biome-scale temperature sensitivity of ecosystem respiration revealed by atmospheric CO_2_ observations. Nat Ecol Evol 2023; 7: 1199–210. 10.1038/s41559-023-02093-x PMC10406605 37322104

[16] Qin S, Kou D, Mao C et al. Temperature sensitivity of permafrost carbon release mediated by mineral and microbial properties. Sci Adv 2021; 7: eabe3596. 10.1126/sciadv.abe3596 PMC8346221 34362729

[17] Sáez-Sandino T, García-Palacios P, Maestre FT et al. The soil microbiome governs the response of microbial respiration to warming across the globe. Nat Clim Change 2023; 13: 1382–7. 10.1038/s41558-023-01868-1

[18] Dutta K, Schuur E, Neff J et al. Potential carbon release from permafrost soils of Northeastern Siberia. Glob Change Biol 2006; 12: 2336–51. 10.1111/j.1365-2486.2006.01259.x

[19] Gentsch N, Wild B, Mikutta R et al. Temperature response of permafrost soil carbon is attenuated by mineral protection. Glob Change Biol 2018; 24: 3401–15. 10.1111/gcb.14316 29774972

[20] Ding J, Chen L, Zhang B et al. Linking temperature sensitivity of soil CO_2_ release to substrate, environmental, and microbial properties across alpine ecosystems. Glob Biogeochem Cycles 2016; 30: 1310–23. 10.1002/2015GB005333

[21] Schuur EA, Abbott BW, Commane R et al. Permafrost and climate change: carbon cycle feedbacks from the warming Arctic. Annu Rev Environ Resour 2022; 47: 343–71. 10.1146/annurev-environ-012220-011847

[22] Pei J, Fang C, Li B et al. Direct evidence for microbial regulation of the temperature sensitivity of soil carbon decomposition. Glob Change Biol 2024; 30: e17523. 10.1111/gcb.17523 39377428

[23] Ren S, Ding J, Yan Z et al. Higher temperature sensitivity of soil C release to atmosphere from northern permafrost soils as indicated by a meta-analysis. Glob Biogeochem Cycles 2020; 34: e2020GB006688. 10.1029/2020GB006688

[24] Tang X, Zhao X, Bai Y et al. Carbon pools in China’s terrestrial ecosystems: new estimates based on an intensive field survey. Proc Natl Acad Sci USA 2018; 115: 4021–6. 10.1073/pnas.1700291115 PMC5910800 29666314

[25] Wang J, Liu D. Models overestimate ecosystem water use efficiency for northern permafrost regions. Agric For Meteorol 2023; 339: 109594. 10.1016/j.agrformet.2023.109594

[26] Haaf D, Six J, Doetterl S. Global patterns of geo-ecological controls on the response of soil respiration to warming. Nat Clim Change 2021; 11: 623–7. 10.1038/s41558-021-01068-9

[27] Craine JM, Fierer N, McLauchlan KK. Widespread coupling between the rate and temperature sensitivity of organic matter decay. Nat Geosci 2010; 3: 854–7. 10.1038/ngeo1009

[28] Kayler Z, Kaiser M, Gessler A et al. Application of δ^13^C and δ^15^N isotopic signatures of organic matter fractions sequentially separated from adjacent arable and forest soils to identify carbon stabilization mechanisms. Biogeosciences 2011; 8: 2895–906. 10.5194/bg-8-2895-2011

[29] Rowley MC, Grand S, Verrecchia ÉP. Calcium-mediated stabilisation of soil organic carbon. Biogeochemistry 2018; 137: 27–49. 10.1007/s10533-017-0410-1

[30] Wen S, Hu A, Jiang S et al. Temperature sensitivity of organic carbon decomposition in lake sediments is mediated by chemodiversity. Glob Change Biol 2024; 30: e17158. 10.1111/gcb.17158

[31] Conant RT, Drijber RA, Haddix ML et al. Sensitivity of organic matter decomposition to warming varies with its quality. Glob Change Biol 2008; 14: 868–77. 10.1111/j.1365-2486.2008.01541.x

[32] Xu M, Li X, Kuyper TW et al. High microbial diversity stabilizes the responses of soil organic carbon decomposition to warming in the subsoil on the Tibetan Plateau. Glob Change Biol 2021; 27: 2061–75. 10.1111/gcb.15553 33560552

[33] Gutiérrez-Girón A, Díaz-Pinés E, Rubio A et al. Both altitude and vegetation affect temperature sensitivity of soil organic matter decomposition in Mediterranean high mountain soils. Geoderma 2015; 237–238: 1–8. 10.1016/j.geoderma.2014.08.005

[34] Sazykina M, Minkina T, Konstantinova EY et al. Pollution impact on microbial communities composition in natural and anthropogenically modified soils of Southern Russia. Microbiol Res 2022; 254: 126913. 10.1016/j.micres.2021.126913 34798540

[35] Eichorst SA, Kuske CR, Schmidt TMJA et al. Influence of plant polymers on the distribution and cultivation of bacteria in the phylum *Acidobacteria*. Appl Environ Microb 2011; 77: 586–96. 10.1128/AEM.01080-10 PMC3020536 21097594

[36] Dong X, Liu C, Ma D et al. Organic carbon mineralization and bacterial community of active layer soils response to short-term warming in the Great Hing’an Mountains of Northeast China. Front Microbiol 2021; 12: 802213. 10.3389/fmicb.2021.802213 PMC8739994 35003032

[37] Wang C, Morrissey EM, Mau RL et al. The temperature sensitivity of soil: microbial biodiversity, growth, and carbon mineralization. ISME J 2021; 15: 2738–47. 10.1038/s41396-021-00959-1 PMC8397749 33782569

[38] Li Y, Wang J, Shen C et al. Microbial diversity losses constrain the capacity of soils to mitigate climate change. Glob Change Biol 2024; 30: e17601. 10.1111/gcb.17601 39624954

[39] García FC, Clegg T, O’Neill DB et al. The temperature dependence of microbial community respiration is amplified by changes in species interactions. Nat Microbiol 2023; 8: 272–83. 10.1038/s41564-022-01283-w 36732470

[40] Yuan MM, Guo X, Wu L et al. Climate warming enhances microbial network complexity and stability. Nat Clim Change 2021; 11: 343–8. 10.1038/s41558-021-00989-9

[41] Qin S, Zhang D, Wei B et al. Dual roles of microbes in mediating soil carbon dynamics in response to warming. Nat Commun 2024; 15: 6439. 10.1038/s41467-024-50800-4 PMC11291496 39085268

[42] Fuhrman JA . Microbial community structure and its functional implications. Nature 2009; 459: 193–9. 10.1038/nature08058 19444205

[43] Romaní AM, Fischer H, Mille-Lindblom C et al. Interactions of bacteria and fungi on decomposing litter: differential extracellular enzyme activities. Ecology 2006; 87: 2559–69. 10.1890/0012-9658(2006)87[2559:iobafo]2.0.co;2 17089664

[44] Li W, Gao X, Du L et al. Responses of *Q*_10_ to anthropogenic soil redistribution: synthetic effects of SOM composition and r/K-shifts of microbial community. Soil Tillage Res 2022; 223: 105477. 10.1016/j.still.2022.105477

[45] Anthony MA, Crowther TW, Maynard DS et al. Distinct assembly processes and microbial communities constrain soil organic carbon formation. One Earth 2020; 2: 349–60. 10.1016/j.oneear.2020.03.006

[46] Li J, Pei J, Fang C et al. Thermal adaptation of microbial respiration persists throughout long-term soil carbon decomposition. Ecol Lett 2023; 26: 1803–14. 10.1111/ele.14296 37592863

[47] Huang Q, Wang B, Shen J et al. Shifts in C-degradation genes and microbial metabolic activity with vegetation types affected the surface soil organic carbon pool. Soil Biol Biochem 2024; 192: 109371. 10.1016/j.soilbio.2024.109371

[48] Li H, Yang S, Semenov MV et al. Temperature sensitivity of SOM decomposition is linked with a K-selected microbial community. Glob Change Biol 2021; 27: 2763–79. 10.1111/gcb.15593 33709545

[49] Chen Y, Liu F, Kang L et al. Large-scale evidence for microbial response and associated carbon release after permafrost thaw. Glob Change Biol 2021; 27: 3218–29. 10.1111/gcb.15487 33336478

[50] Keiluweit M, Bougoure JJ, Zeglin LH et al. Nano-scale investigation of the association of microbial nitrogen residues with iron (hydr) oxides in a forest soil O-horizon. Geochim Cosmochim Acta 2012; 95: 213–26. 10.1016/j.gca.2012.07.001

[51] Liu F, Qin S, Fang K et al. Divergent changes in particulate and mineral-associated organic carbon upon permafrost thaw. Nat Commun 2022; 13: 5073. 10.1038/s41467-022-32681-7 PMC9424277 36038568

[52] Davidson EA, Janssens IA. Temperature sensitivity of soil carbon decomposition and feedbacks to climate change. Nature 2006; 440: 165–73. 10.1038/nature04514 16525463

[53] Qin S, Fang K, Song Y et al. Linkage between temperature sensitivity of SOM decomposition and microbial communities depends on soil fractions. Glob Change Biol 2024; 30: e17456. 10.1111/gcb.17456 39109396

[54] Zhou Z, Ren C, Wang C et al. Global turnover of soil mineral-associated and particulate organic carbon. Nat Commun 2024; 15: 5329. 10.1038/s41467-024-49743-7 PMC11193739 38909059

[55] Jia N, Li L, Guo H et al. Important role of Fe oxides in global soil carbon stabilization and stocks. Nat Commun 2024; 15: 10318. 10.1038/s41467-024-54832-8 PMC11605054 39609446

[56] Wang C, Pan X, Yu W et al. Aridity and decreasing soil heterogeneity reduce microbial network complexity and stability in the semi-arid grasslands. Ecol Indic 2023; 151: 110342. 10.1016/j.ecolind.2023.110342

[57] Yuste JC, Penuelas J, Estiarte M et al. Drought-resistant fungi control soil organic matter decomposition and its response to temperature. Glob Change Biol 2011; 17: 1475–86. 10.1111/j.1365-2486.2010.02300.x

[58] Han M, Chen Y, Sun L et al. Linking rhizosphere soil microbial activity and plant resource acquisition strategy. J Ecol 2023; 111: 875–88. 10.1111/1365-2745.14067

[59] Wang C, Yu Q, Ji N et al. Bacterial genome size and gene functional diversity negatively correlate with taxonomic diversity along a pH gradient. Nat Commun 2023; 14: 7437. 10.1038/s41467-023-43297-w PMC10656551 37978289

[60] Celi L, Presta M, Ajmore-Marsan F et al. Effects of pH and electrolytes on inositol hexaphosphate interaction with goethite. Soil Sci Soc Am J 2001; 65: 753–60. 10.2136/sssaj2001.653753x

[61] Smith LC, Orgiazzi A, Eisenhauer N et al. Large-scale drivers of relationships between soil microbial properties and organic carbon across Europe. Glob Ecol Biogeogr 2021; 30: 2070–83. 10.1111/geb.13371

[62] Jia J, de Goede R, Li Y et al. Unlocking soil health: are microbial functional genes effective indicators? Soil Biol Biochem 2025; 204: 109768. 10.1016/j.soilbio.2025.109768

[63] Gao Y, Yu Z, Zhou M et al. Optimal reliability ensemble averaging approach for robust climate projections over China. Int J Climatol 2024; 44: 2852–75. 10.1002/joc.8485

[64] Bradford MA, Wieder WR, Bonan GB et al. Managing uncertainty in soil carbon feedbacks to climate change. Nat Clim Change 2016; 6: 751–8. 10.1038/nclimate3071

[65] Guo X, Gao Q, Yuan M et al. Gene-informed decomposition model predicts lower soil carbon loss due to persistent microbial adaptation to warming. Nat Commun 2020; 11: 4897. 10.1038/s41467-020-18706-z PMC7524716 32994415

[66] Wieder WR, Bonan GB, Allison SD. Global soil carbon projections are improved by modelling microbial processes. Nat Clim Change 2013; 3: 909–12. 10.1038/nclimate1951

[67] Fang K, Chen L, Qin S et al. Mineral and climatic controls over soil organic matter stability across the Tibetan alpine permafrost region. Glob Biogeochem Cycles 2021; 35: e2021GB007118. 10.1029/2021GB007118

[68] Brown J, Ferrians O, Heginbottom J et al. Circum-Arctic Map of Permafrost and Ground-Ice Conditions, Version 2. Boulder, Colorado: National Snow and Ice Data Center, 2002.

[69] Obu J, Westermann S, Bartsch A et al. Northern Hemisphere permafrost map based on TTOP modelling for 2000–2016 at 1 km^2^ scale. Earth Sci Rev 2019; 193: 299–316. 10.1016/j.earscirev.2019.04.023

[70] Zou D, Zhao L, Sheng Y et al. A new map of permafrost distribution on the Tibetan Plateau. Cryosphere 2017; 11: 2527–42. 10.5194/tc-11-2527-2017

