# Supplementary material for: Heterogeneous response of soil organic matter decomposition to temperature change across permafrost regions
Source: Natl Sci Rev. 2026 Jan 12;13(20):nwag019. doi: 10.1093/nsr/nwag019 (PMC13767798; doi:10.1093/nsr/nwag019)
Supplement: Supplementary file 1 [file nwag019_supplemental_files.zip › Yang et al. SI.docx]

**Supplementary Data for**

**Heterogeneous response of soil organic matter decomposition to temperature change across permafrost regions**

Yan Yang^1,2^, Shuqi Qin^1,2^, Tadeo Sáez-Sandino^3^, Leiyi Chen^1,2^, Siyu Wang^1,2,4^, Pete Smith^5^, Luyao Kang^1,2^, and Yuanhe Yang^1,2,4*^

^1^State Key Laboratory of Forage Breeding-by-Design and Utilization; Key Laboratory of Vegetation and Environmental Change, Institute of Botany, Chinese Academy of Sciences, Beijing, China.

^2^China National Botanical Garden, Beijing, China.

^3^Departamento de Sistemas Físicos, Químicos y Naturales, Universidad Pablo de Olavide, Seville, Spain.

^4^University of Chinese Academy of Sciences, Beijing, China.

^5^Institute of Biological and Environmental Sciences, University of Aberdeen, Aberdeen, UK.

***Corresponding author:** Prof. Yuanhe Yang, tel.: +86-10-6283 6638, E-mail: yhyang@ibcas.ac.cn

**Supplementary Data**

Methods

Supplementary Notes 1 to 4

Figures S1 to S17

Tables S1 to S5

Supplementary Reference lists 1 to 2

**METHODS**

**Study area**

As representatives of high-altitude and high-latitude permafrost regions, the Tibetan Plateau (32°34′ ~ 38°34′ N, 91°51 ′N ~ 99°30′ E, 3774-5078 m elevation) and the Northeast China (47°37′ ~ 53°26′ N, 117°32′ N ~ 129°22′ E, 300-895 m elevation) were chosen as the study areas (Fig. 1a-c). Among them, the Tibetan Plateau features a cold semi-arid climate, with a mean annual temperature (MAT) ranging from -4.5 and 1.8 ℃ and mean annual precipitation (MAP) between 245 and 504 mm. The active layer thickness on the plateau averages 1.9 m [1], and the soil organic carbon (C) density (SOCD, C amount per area) in this region is 18.3 kg C m^-2^ at 1 m depth [2]. Vegetation predominantly includes alpine steppe, alpine meadow, and swamp meadow [3]. Specifically, alpine steppe is predominantly composed of *Stipa purpurea* and *Carex moorcroftii,* while alpine meadow is mainly dominated by *Kobresia pygmaea* and *K. humilis* [4]*.* Additionally*,* swamp meadow is primarily characterized by *K. tibetica* [4]. Soil types in the area are categorized as Cambisol, Calcisol, and Cryosols [5]. The SOCD is highest in swamp meadow, followed by alpine meadow, and lowest in alpine steppe [6].

The permafrost region in Northeast China is situated in a cold temperate semi-humid climate zone, along the southern edge of the Eurasian permafrost boundary. The MAT and MAP range from -4.3 to 1.7 ℃ and 236 to 680 mm, respectively. Given that the low MAT in this region was predominantly determined by its latitude rather than by elevation [7], the permafrost region in Northeast China has been widely classified as the southern edge of high-latitude permafrost region [8,9]. The SOCD in this region averages 31.4 kg C m^-2^ at 1 m depth [2]. The active layer thickness ranges from 1.6 to 1.8 m in more than half of the areas (55%), with a mean value of 1.7 m [10]. Vegetation primarily consists of deciduous coniferous and mixed forests, with a small proportion of grasslands. The arbor layer is dominated by *Larix gmelini*, mixed with small amounts of *Betula platyphylla* and *Quercus mongolica*. Grasslands are dominated by *Leymus chinensis*, *S. grandis*, and *Artemisia frigida*. The primary soil type in this region is Cambisol [5].

**Field sampling**

To investigate *Q*_10_ differences and their driving factors, we conducted two regional surveys in the permafrost areas of the Tibetan Plateau and Northeast China. In 2016, 24 sampling sites (15 selected for this study) were established along a 1,000-km grassland transect which covers alpine steppe, alpine meadow and swamp meadow on the plateau and also encompasses both sporadic and discontinuous permafrost zones [11]. In 2023, 30 sampling sites, 15 of which were chosen for this study, were investigated in the permafrost region of Northeast China, which comprises forest and grassland ecosystems and also includes isolated and discontinuous permafrost zones (Table S1). These selected sites span a broad environmental gradient, consistent with the environments represented by 24 sampling locations on the Tibetan Plateau and 30 sampling sites in Northeast China (Fig. S11). The MAT was at or below 0℃ at nearly all sampling sites (Table S1). On the Tibetan Plateau, one square plot (10 m × 10 m) was randomly arranged at each grassland site, with five quadrats (1 m × 1 m) were set up at the four corners and the center. Soil samples were then collected from surface layer (0-10 cm) and uppermost permafrost samples (50 cm thickness) [11]. In Northern China, the grassland plots followed the same sampling design as those on the Tibetan Plateau. For forest ecosystem, three 20 m × 30 m plots were established at each site and soil samples were collected along the diagonals of each plot. Soil samples were then obtained from depths of 0-10, 10-20, 20-30 and 30-50 cm [12]. For detailed sampling procedures, refer to ref. [12]. Given the high C content and microbial activity in the topsoil [13], samples from the 0-10 cm depth were selected for analysis in both regions.

To manage experimental cost, we selected 15 sites from 24 sampling sites on the Tibetan Plateau, comprising 9 alpine meadows, three alpine steppes, and three swamp meadows. Similarly, 15 sites were chosen from 30 sampling sites in permafrost region of the Northeast China, including 12 forests and three grasslands. Soil samples from the same layer at each plot were mixed evenly as a composite sample to reduce spatial heterogeneity. Afterward, part of the composite sample was stored at -20°C until laboratory incubation. while another portion was naturally air-dried to remove roots, gravel, etc., and then used to determine soil physicochemical properties. Before incubation, the composite samples were pre-incubated at 15°C and 60% of the field water capacity, followed by microbial analysis.

***Q*_10_ measurements based on laboratory incubation**

**Incubation experiment.** For each sampling site, we measured soil C release and calculated *Q*_10_ by laboratory microcosms with fresh soil samples being aerobically incubated at 5 and 15℃. The selection of incubation temperatures referred to previous studies conducted in permafrost regions [14-16] and also corresponded to the average peak growing-season (July–August) temperatures on the Tibetan Plateau and in Northeast China, which were approximately 5 °C and 15 °C, respectively (Fig. S12). In each microcosm, 15-25 g of homogenized fresh soil was weighed into a 250 ml jar and pre-incubated at 15℃ for 7 days [1,17] to reactivate microbial activity. During incubation, deionized water was added to sustain soil moisture at 60% of the field water capacity. Prior to each gas collection, the jars were sealed and incubated at 5℃ or 15℃ [14,16] for 20 h after being purged with synthetic air (80% N_2_ and 20% O_2_) for 8 min. Afterwards, headspace gas samples were collected using 10 ml syringes on days 1, 3, 6, 10, 14, 19, 25, 31, 41, 49, 70, 86, 102, 118, 141, 173, 218, and 368, spanning a total of 368 days. This long-term incubation was designed to capture the wide dynamic range of soil organic matter (SOM) decomposition and to reduce instability in soil respiration rates arising from the rapid depletion of labile C pools [18]. CO_2_ concentrations were determined using a portable infrared gas analyzer (EGM-5; PP Systems, Amesbury, MA, USA). The CO_2_ release rate (R_CO2,_ μg CO_2_-C g^-1^ soil h^-1^) was calculated based on the amount of CO_2_ released per unit mass of soil during the sealing period. Across the incubation, R_CO2_ generally declined over time, and this trend was more pronounced at 15℃ than at 5℃ (Fig. S13a).

***Q*_10_ calculation.** The *Q*_10_ of R_CO2_ was calculated based on the ‘equal time’ method [19] according to Eq. (1).

$Q_{10}=\left( R_{CO_{2}}^{T_{a}}/R_{CO_{2}}^{T_{b}} \right)^{10/T_{a}-T_{b}}$	(1)

where $R_{CO_{2}}^{T_{a}}$ and $R_{CO_{2}}^{T_{b}}$ represent the CO_2_ release rate at 15℃ and 5℃ (μg CO_2_-C g^-1^ soil h^-1^), respectively. $T_{a}$ and $T_{b}$correspond to the warmer and cooler temperatures (°C). The *Q*_10_ value was then calculated as the average of the 18 measurements taken throughout the incubation period. Considering substrate consumption might influence the temperature response of SOM decomposition, we further calculated the *Q*_10-q_ values using ‘equal-C’ method [20], which estimated temperature sensitivity under equivalent levels of substrate depletion through the following two steps. (i) A first-order kinetic one-compartment model (Eq. 2) was applied to fit the relationship between temporal changes in cumulative C mineralization and time at each site [21].

$C_{Cum}\left( t \right)=C_{0}\left( 1-e^{-k_{0}t} \right)$	(2)

where $C_{Cum}\left( t \right)$is cumulative C mineralized up to time $t$(mg CO_2_-C g^-1^ OC), $C_{0}$ is the potential mineralizable C (mg CO_2_-C g^-1^ OC), *k_0_* is decomposition rate constant (day^-1^), and *t* is time (day). (ii) We estimated the *Q*_10-q_ values by comparing the time required to mineralize an equivalent amount of SOM [20]. Across all sites, the cumulative respired soil C at the lower temperature (5℃) after 368 days of incubation ranged from 0.9-2.3% in Northeast China to 2.7-15.9% on the Tibetan Plateau (Fig. S13b). Therefore, we calculated *Q*_10-q_ values at cumulative respiratory soil C levels of 0.2-0.4% and 0.6-0.8%, reflecting a gradient of progressively diminishing substrate availability (Eq. 3).

$Q_{10-q}=\left( t_{b}/t_{a} \right)^{\left[ 10/\left( T_{a}-T_{b} \right) \right]}$	(3)

Here, $t_{a}$ and$t_{b}$ represent the times (days) taken to respire a given fraction of soil organic carbon (SOC) at the higher temperature (15°C) and at the lower temperature (5°C), respectively, $T_{a}$ and $T_{b}$correspond to the warmer and cooler temperatures (°C).

**Climate and edaphic properties**

To investigate the potential effects of climate and soil variables on soil *Q*_10_ variations across permafrost regions, we used multiple climate datasets. Specifically, MAT ([https://data.tpdc.ac.cn/zh-hans/data/71ab4677-b66c-4fd1-a004-b2a541c4d5bf](about:blank)) and MAP (https://data.tpdc.ac.cn/zh-hans/data/faae7605-a0f2-4d18-b28f-5cee413766a2) were obtained for the period 1982-2011 from the National Tibetan Plateau/Third Pole Environment Data Center (https://data.tpdc.ac.cn/home) based on the geographic coordinates of sampling sites. Aridity index, defined as the ratio of precipitation to potential evapotranspiration [22], was extracted from the Global Aridity Index and Potential Evapotranspiration Climate database (<https://cgiarcsi.community/>), where lower aridity index indicates higher aridity. For easier understanding, we used 1-aridity index to represent aridity severity [23]. For edaphic properties, soil pH was measured using a pH meter (PB-10; Sartorius, Göttingen, Germany) with a water: soil ratio of 2.5:1 (volume/weight). Soil texture was assessed with the Masterizer 2000 particle size analyzer (Malvern, Worcestershire, UK) following the removal of SOM (30% H_2_O_2_) and carbonates (30% HCl) from samples.

**Substrate quantity and quality**

For substrate quantity, SOC concentration was measured using a Vario EL Ⅲ element analyzer (Multi EA 4000, Analytik Jena, Jena, Germany) after eliminating inorganic C by adding 1 M HCl to the soil when it was alkaline. The molecular structure of SOM was established using solid-state cross-polarization /magic angle spinning ^13^C nuclear magnetic resonance spectroscopy (^13^C CP/MAS NMR). Air-dried soil samples were regularly preprocessed with 10% hydrofluoric acid to eliminate paramagnetic compounds (*e.g.*, Fe^3+^, and Mn^2+^) and enhance NMR sensitivity [24]. The ^13^C NMR spectra were acquired with an AVANCE NeO 600 MHz spectrometer (Bruker BioSpin, Rheinstetten, Baden-Württemberg, Germany), featuring a CP/MAS probe and operating at a Larmor frequency of 100.6 MHz. Air-dried and powdered samples were packed into a zirconium dioxide rotor (OD = 4 mm) with an 8 kHz spinning rate, a 2.5 ms contact time, and a 2.0 s recycle delay. The ^13^C cross polarization/total sideband suppression (CP/TOSS) technique was employed to identify functional groups within SOM. Spectral quantification and baseline correction were performed using MestReNova 9.0 (Mestrelab Research S.L., Santiago de Compostela, Galicia, Spain) [25]. The NMR spectra were classified into four chemical groups: alkyl C (0–50 ppm), O-alkyl C (50–110 ppm), aromatic C (110–165 ppm), and carbonyl C (165–220 ppm) (Fig. S14). Finally, we characterized substrate quality by analyzing the relative abundances of O-alkyl and aromatic C, as well as alkyl/O-alkyl ratio, with the higher ratio indicating the lower substrate quality [26].

**Mineral protection**

To clarify the effects of mineral protection on soil *Q*_10,_ we characterized the adsorption potential of minerals on SOC by examining the proportions of mineral-associated organic carbon (MAOC), iron-bound carbon (OC-Fe), and calcium-bound organic C (OC-Ca). First, the relative contribution of MAOC to the total SOC was assessed using a fractionation method that integrates particle size and density analyses [27]. Specifically, 10 g of dry soil was ultrasonically dispersed in a 1.85 g cm^-3^ NaI solution. The suspension was then centrifuged at 1800 × g, and filtered through a GF/C filter membrane (Whatman GmbH, Dassel, Germany). The fraction retained on the filter was collected as light particulate organic matter (fPOM, <1.85 g cm^-3^). The remaining fraction was rinsed onto a 53-μm sieve, with the material passing through the sieve was collected as MAOM, while the retained fraction was collected as heavy particulate organic matter (hPOM). All collected components were dried at 60℃, achieving an average mass recovery of 94.5 ± 0.5%. After the elimination of inorganic C by 1 M HCl, the C content of MAOM samples was measured using a Vario EL Ⅲ elemental analyzer (Multi EA 4000, Analytik Jena, Jena, Germany).

Second, the OC-Fe content was determined by the citrate-bicarbonate-dithionite (CBD) method [28]. Briefly, two identical 0.25 g of air-dried soil were prepared. The treatment sample was extracted with a CBD solution (0.1 M sodium dithionite + 0.11 M sodium bicarbonate + 0.27 M trisodium citrate) in a water bath at 80℃, while the control sample was treated with 1.8 M sodium chloride and 0.11 M sodium bicarbonate. The C content of soil residues was measured using a Vario EL Ⅲ elemental analyzer (Multi EA 4000, Analytik Jena, Jena, Germany). The OC-Fe content was calculated as the difference in C content between the treatment and control samples. Finally, OC-Ca was quantified by extraction with 0.5 M Na_2_SO_4,_ followed by measuring the C content in the extract using a multi-N/C 3100 analyzer (Analytik Jena, Jena, Germany) [29].

**Microbial properties**

**DNA extraction and 16S rRNA gene sequencing**. To assess the potential role of microorganisms in mediating soil *Q*_10,_ we analyzed a set of microbial properties including microbial community composition, α diversity, life history strategies, and functional genes. Soil DNA was extracted from 0.2 g of soil with DNeasy PowerSoil Pro Kits (Qiagen, Hilden, Germany) in accordance with the manufacturer’s protocol. DNA quality was checked using a NanoDrop 2000 spectrophotometer (Thermo Fisher Scientific, Waltham, MA, USA) by determining the absorbance ratios at 260/230 nm and 260/280 nm. For bacteria, the V3-V4 region of 16S rRNA gene was amplified with the primer pair 338F (5'-ACTCCTACGGGAGGCAGCA-3') and 806R (5'- GGACTACHVGGGTWTCTAAT -3') [30]. For fungi, primers ITS3 (5′-GCATCGATGAAGAACGCAGC-3′) and ITS4 (5′-TCCTCCGCTTATTGATATGC-3′) were used to amplify ITS2 rRNA gene hypervariable region [31]. The PCR conditions for amplifying the 16S rRNA gene were as follows: initial denaturation at 94℃ for 5 min, followed by 31 cycles of denaturation at 94℃ for 30 s, annealing at 53℃ for 30 s, extension at 72℃ for 30 s, and final elongation at 72℃ for 8 min. The thermocycling of ITS2 rRNA was: 3 min at 95℃ for initialization, 35 cycles of 20 s denaturation at 95℃, 20 s annealing at 56℃, 30 s extension at 72℃, and 5 min final extension at 72℃. The concentration and length of the PCR products were determined using 1.5% agarose gel electrophoresis. Sequencing libraries were prepared using the TruSeqTM DNA Sample Prep Kit (New England Biolabs, MA, USA), and assessed with a Qubit 4.0 Fluorometer (Thermo Fisher Scientific, MA, USA). DNA extracts were sequenced on the Illumina Nova6000 platform (Guangdong Magigene Biotechnology Co., Ltd. Guangzhou, China).

Raw sequences were evaluated for quality using FastQC v.0.12.0 [32]. Paired-end sequences were then merged by USEARCH v10.0.240 [33], and primers and barcodes were trimmed based on VSEARCH v2.15.2 [34]. Subsequently, ASVs with 100% similarity were obtained using the unoise3 algorithm [35] in USEARCH. Taxonomical annotation of representative ASVs was performed with SILVA138 database ([https://www.arb-silva.de/](about:blank)) for bacteria and UNITE v7.1 database ([https://unite.ut.ee/index.php](about:blank)) for fungi, with ASVs identified as non-bacteria or non-fungi being excluded from the following analyses. To maintain uniform sampling depth within each sample, the ASV abundance table was normalized at the minimal sequence per sample.

**Microbial community structure and life history strategy analyses.** To examine dissimilarities in microbial community structure between two permafrost regions, principal coordinate analysis (PCoA) based on Bray-Curtis distance was performed with the “Vegan” package [36] in R v4.3.2 [37], and Permutational multivariate analysis of variance (PERMANOVA) was also conducted with the same package. The liner discriminant analysis (LDA) Effect Size (LEfse) was constructed to identify enriched species at multiple taxonomical levels [38], with an LDA significance threshold of > 4 and a *P* value cutoff of 0.01 in the Kruskal-Wallis rank sum test [39]. Microbial α diversity (Shannon index) was calculated based on the “Vegan” package [36] in R v4.3.2 [37].

For assessing bacterial life history strategies, the ribosomal RNA gene operon (*rrn*) copy number serves as a key indicator of bacterial life history [40]. It is generally believed that bacteria with multiple *rrn* operon copy numbers correspond to r-strategists, while those with fewer *rrn* operons are more aligned with K-strategists [41]. The *rrn* copy numbers were estimated by consulting the closest known rRNA operon copy number with the rrnDB v5.4 database (https://rrndb. umms.med.umich.edu/) [42]. The community-level *rrn* copy was calculated by multiplying the *rrn* copy number of each ASV by its relative abundance and then summing these values across all ASVs in the sample using Eq. (4) as follows [43]:

$Community-level rrn copy number=\sum_{i=1}^{n} (rrn_{i}\times S_{i})$	(4)

where *n* is numbers of ASVs, *rrn_i_* is the estimated *rrn* copy number of ASV_i,_ *S_i_* denotes the abundance of ASV_i._

To evaluate fungal life history strategies, fungal guilds were classified into trophic types (Saprotroph, Symbiotroph, and Pathotroph) by FUNGuild [44]. Saprotrophic fungi, which thrive in nutrient-rich environments, are categorized as r-strategists [45,46]. Conversely, symbiotrophic fungi (*e.g.*, ectomycorrhizal fungi) exhibit slower growth rates, are adapted to nutrient-poor environments, and play a pivotal role in the mineralization of recalcitrant SOM, thus classifying them as K-strategists [45,46]. Based on this understanding, we quantified fungal K/r strategies by calculating the ratio of symbiotrophic to saprotrophic fungi (Sym/Sap) [45].

**Measurement of functional gene abundances.** To evaluate the functional potential of microbial C decomposition, we employed the high-throughput qPCR-based chip (QMEC) technique [47] to determine the absolute abundance of functional genes involved in C degradation. The targeted genes included those associated with the degradation of starch (*apu* and *sga*), hemicellulose (*xylA*, *abfA*, and *manB*), cellulose (*cdh* and *cex*), chitin (*chiA*), pectin (*pgu*), and lignin (*glx*, *lig*, and *mnp*) (Table S5). Amplification was performed on SmartChip Real-Time PCR System (WaferGen Biosystems, Fremont, USA). Briefly, the qPCR protocol consisted of an initial denaturation at 95℃ for 5 min, followed by 40 cycles of 95℃ for 15 s, 58℃ for 30 s, and extension at 72℃ for 30 s. To ensure reproducibility, each sample was amplified in triplicate, and each run contained a negative control. The 16S rRNA gene was used as the internal reference gene to ensure reliable quantitative comparisons among samples. In this study, the gene copy number of 16S rRNA per unit of soil was estimated based on the sample weight and the total amount of extracted DNA.

**Synthesis of *Q_10_* measurements across Arctic permafrost region**

To compare with observations from Arctic permafrost regions, we compiled a *Q*_10_ dataset derived from previously published studies across Arctic permafrost regions. A literature search was conducted in the Web of Science (<https://webofscience.clarivate.cn>) and Google Scholar (https://scholar.google.com) using the keywords “temperature sensitivity or temperature response” and “soil C decomposition or microbial respiration”, covering publications available up to October 2025. The individual studies were included based on the following criteria: (i) reported *Q*_10_ values; (ii) provided geographic coordinates within the Circum-Arctic map of permafrost [48]; (iii) specified the sampled soil horizon, with data only from the organic topsoil being retained; and (iv) incubation temperatures were consistent with those used in this study (5°C and 15°C). In total, six individual studies (see Supplementary References List 1) were incorporated into the final *Q*_10_ dataset.

**Statistical analyses**

We analyzed the data in three steps. First, the Wilcoxon rank-sum test was employed to compare climate variables (MAT, MAP, and aridity), soil properties (pH, clay + silt), substrate quantity (SOC) and quality (O-alkyl C, aromatic C, and alkyl/O-alkyl ratio), mineral protection (proportions of MAOC, OC-Fe, and OC-Ca), microbial attributes (community structure, α diversity, life history strategies, and C-degradation genes), and *Q*_10_ values between high-altitude and high-latitude permafrost regions. The Kruskal–Wallis rank sum test, followed by Dunn’s post hoc test [49], was performed for non-parametric data to determine significant differences among various permafrost types. In subsequent analyses, data were log-transformed when necessary.

Second, Spearman and partial correlations were conducted to assess the influences of

environmental factors (climate and soil properties, including aridity, soil texture and pH), substrate properties (SOC, O-alkyl C, aromatic C, and alkyl/O-alkyl ratio), mineral protection (the proportion of SOC associated with MAOC, OC-Fe, and OC-Ca), and soil microbiome (community composition, α diversity, life history strategies, and C-degrading genes) on *Q*_10_ across the two permafrost regions, and the “fdr” adjustment method [50] was applied to control the likelihood of false discoveries in multiple testing correlation.

Third, a piecewise SEM was developed to further clarify the direct and indirect effects of climate, soil properties, substrate quantity, mineral protection, and microbial properties on *Q*_10_ variations, guided by a priori model (Fig. S15). Specifically, we hypothesized that (1) mineral protection directly influences soil *Q*_10_ by restricting substrate accessibility through organic–mineral associations; (2) soil microorganisms, as the ultimate agents of SOM decomposition, directly affect *Q*_10_; (3) climate, soil properties, and substrate characteristics influence *Q*_10_ either directly or indirectly by modulating mineral protection and microbial properties. Given that laboratory experiment was conducted under constant soil temperatures and moisture, we took climate factors as the representative of the legacy effect, that is, the direct influence of climate variables on *Q*_10_ was not included in the prior model.

In the subsequent SEM analysis, only factors showing significant differences between the two permafrost regions and having notable correlations with soil *Q*_10_ were included. To minimize collinearity among factors within each group, PCA was used to generate composite indicators for soil conditions and microbial properties. Among climate variables, MAT was not chosen because it did not differ significantly between the two regions, and aridity was selected to represent water conditions because of its high collinearity with MAP. After assessing the collinearity within each group of variables, which showed a lack of multicollinearity (Table S3), the PC1 axes of soil and microbial attributes were chosen for analysis. Within each group, PCA explained more than 60% of the variance (Table S2). The fitness of the final model was evaluated using Fisher's C statistic, the Akaike information criterion (AIC), and *P* values. All statistical analyses were performed using R v4.3.2 [37].

**Supplementary Note 1 Variations of soil *Q*_10_ among different permafrost types.**

Our results showed that *Q*_10_ values in isolated permafrost regions were significantly higher than in discontinuous and sporadic permafrost areas (*P* < 0.05, Fig. S2). Such a pattern may be due to the situation that isolated permafrost is generally considered to develop a thicker active layer accompanied by higher water availability [51,52], which may facilitate the diffusion of soluble organic matter [53-55] and enhance substrate availability for microbial processes. However, our observations revealed that the active layer thickness in isolated permafrost was only significantly greater than in continuous permafrost (*P* < 0.05), without any statistical difference compared with discontinuous or sporadic permafrost areas (All *P* = 0.9, Fig. S16), which did not support this possibility. To further explore the potential reasons for this observed difference, we extracted Enhanced Vegetation Index (EVI) data for the peak growing season (July–August) from 2000 to 2022 (MOD13A3.061; [https://appeears.earthdatacloud.nasa.gov](https://appeears.earthdatacloud.nasa.gov/)/) considering the pronounced differences in vegetation composition among permafrost types [56]. The results revealed that the EVI in isolated permafrost region was significantly higher than that of other permafrost types (*P* < 0.05, Fig. S3), indicating greater vegetation productivity. The stronger vegetation growth, dominated by coniferous and shrubs, likely elevates inputs of recalcitrant litter [57], which in turn contributes to the higher *Q*_10_ values. Nevertheless, further validation of this interpretation is needed in upcoming studies.

**Supplementary Note 2 Differences in climate, soil, substrate, and mineral protection between the permafrost regions of the Tibetan Plateau and Northeast China.**

Our results indicated that most biotic and abiotic factors differed between the two permafrost regions (Fig. 2). Precipitation-related variables (MAP and aridity) were significantly higher in permafrost region of Northeast China compared to permafrost zone on the Tibetan Plateau (All *P* < 0.01, Fig. 2b, c), although MAT showed no significant difference between the two permafrost regions (*P* = 0.5, Fig.2a). In terms of soil properties, the Tibetan alpine permafrost region predominantly featured alkaline soils (8.4 ± 0.2, data was presented as mean ± standard error unless noted), while the permafrost region in Northeast China was characterized by acidic soils (5.5 ± 0.1) (Fig. 2d). Both clay + silt content (*P* < 0.001, Fig. 2e) and SOC content (*P* < 0.05, Fig. 2f) were significantly higher in the permafrost region of Northeast China. The higher SOC content in Northeast China was likely attributable to slow microbial decomposition rates under low temperatures, coupled with higher precipitation that enhances plant productivity and, in turn, promotes SOC buildup [58].

Our results also revealed that soil aromatic C content in the permafrost region of Northeast China was significantly higher than that on the Tibetan Plateau (*P* < 0.001, Fig. 2h), while O-alkyl C showed the opposite trend (*P* < 0.05, Fig. 2g). This might be attributed to the high C/N ratio of plant litter, primarily from deciduous and coniferous forests in Northeast China [59], which promotes the accumulation of aromatic C. However, alkyl/O-alkyl C ratio showed no significant difference between the two regions (*P* = 0.9, Fig. 2i). Regarding mineral properties, no significant differences were observed in either the proportion of OC-Fe (*P* = 0.3, Fig. 2k) or MAOC (*P* = 0.6, Fig. 2j) between the two permafrost regions. However, the proportion of OC-Ca was markedly higher in Tibetan alpine permafrost region (*P* < 0.001, Fig. 2l). This disparity was likely due to the alkaline and calcareous soil conditions prevalent on the Tibetan Plateau [14], where Ca^2+^ played a more prominent role in stabilizing SOM through cation bridging [60].

**Supplementary Note 3 Differences in microbial community structures between the permafrost regions of the Tibetan Plateau and Northeast China.**

There were notable differences between soil microbial communities in permafrost regions of the Tibetan Plateau and Northeast China (Fig. 3a, b). We observed that the relative abundances of the dominant microbial phyla, including the top ten bacterial phyla (comprising 98.6 ± 0.1% of total abundance) and the top five fungal phyla (accounting for 92.2 ± 1.2% of total abundance), differed significantly across contrasting permafrost regions (Fig. 3c, d). For bacterial communities, the dominant phyla in both regions include Actinobacteria (Tibetan Plateau *vs.* Northeast China: 52.3 ± 2.2% *vs.* 20.2 ± 2.6%), Proteobacteria (22.2 ± 1.6% *vs.* 36.8 ±1.7%), Bacteroidetes (7.1 ± 1.6% *vs.* 2.3 ± 0.4%), and Acidobacteria (3.2 ± 0.5% *vs.* 12.7 ± 1.7%) (Fig. 3c). Notably, the relative abundances of Actinobacteria and Bacteroidota in Tibetan alpine permafrost region were significantly higher than those in the permafrost region of Northeast China (all *P* < 0.001, Fig. 3c). Conversely, Proteobacteria, Verrucomicrobiota, and Planctomycetota displayed the opposite trends across the two permafrost regions (all *P* < 0.001, Fig. 3c). Furthermore, LEfSe analysis revealed that Micrococcales (Actinobacteria) and Bacteroidia (Bacteroidota) were significantly enriched in Tibetan alpine permafrost region (Fig. S17a). Generally, Micrococcales are known for their resistance to drought and alkaline stress [61], which may be facilitated by the arid climate and alkaline soil on the plateau. Conversely, Xanthobacteraceae (Proteobacteria), Candidatus Udaeobacter (Verrucomicrobiota), and Acidobacteriaceae (Acidobacteria) were more abundant in the permafrost region of Northeast China (Fig. S17a). These microbes are highly sensitive to soil pH and have acidophilic characteristics [62-64], so the acid soil in the permafrost region of Northeast China likely promote their proliferation.

For fungal communities, the dominant phyla in the permafrost regions of the Tibetan Plateau and Northeast China were Ascomycota and Basidiomycota, accounting for 87.3% and 76.2% of the total sequences, respectively (Fig. 3d). Specifically, Ascomycota was significantly abundant in the Tibetan alpine permafrost region compared to Northeast China (*P* < 0.001), consistent with a previous study reporting that Ascomycota showed higher abundance under high-elevation conditions [65]. In contrast, Mortierellomycota and Mucoromycota were more prevalent in the Northeast China’s permafrost region (all *P* < 0.001, Fig. 3d). LEfSe analysis further revealed that Mortierellaceae (Mortierellomycota) was significantly enriched in the permafrost region of Northeast China (Fig. S17b). Mortierellaceae are generally saprophytic fungi that rely on extracellular enzyme activity to decompose lignin and carboxylic-rich alicyclic molecules [65,66]. The higher aromatic C content in Northeast China (*P* < 0.001, Fig. 2h) may thus promote the enrichment of these fungi.

**Supplementary Note 4 Construction of soil microbial network.**

To assess differences in potential microbial species associations across two permafrost regions, we applied the SparCC method [67] to construct cross-kingdom co-occurrence networks of bacterial and fungal taxa at each region. The SparCC approach accounts for sparse correlations for compositional data in network construction, thereby providing greater combinatorial robustness compared to traditional correlations [68]. This method employs a Bayesian framework to infer the true compositional fractions from the observed sequencing counts. Under the assumption of unbiased sampling during sequencing and a uniform prior, the posterior joint fractions distribution follows a Dirichlet distribution [67]. Based on this probabilistic framework, correlations between amplicon sequence variants (ASVs) were determined over 20 iterations, with pseudo *P-*values (bilateral interval) being estimated through 100 bootstrap iterations [67]. To concentrate on dominant taxa and ensure the reliability of correlation relationships, ASVs with relative abundances below 0.01% were excluded. Only species present in at least half of the samples were retained [69]. In the network, each node corresponds to an ASV, and edges represent strong and statistically significant correlations between nodes. Only edges with a correlation coefficient |r| > 0.8 and *P*-values < 0.001 were included in the network construction. The network was visualized using Gephi 0.10.1 (https://gephi.org/desktop/).

**Fig. S1 Relationships between *Q*_10_ values and *Q*_10-qS_.** (a) Association of *Q*_10_ values with *Q*_10-q_s at cumulative respired soil C in the range of 0.2–0.4%. (b) Association of *Q*_10_ values with *Q*_10-q_ values at cumulative respired soil C in the range of 0.6–0.8%. *Q*_10_ values were calculated using the ratio of soil CO_2_ release rates at warmer and colder temperatures over the same incubation period [19], whereas *Q*_10-q_s were determined by dividing the time taken to respire a given fraction of soil organic carbon (SOC) at 5°C by that at 15°C [20]. TP and NE represent permafrost regions of the Tibetan Plateau and Northeast China, respectively. The solid line denotes the significant linear regression, and the grey shaded area is 95% confidence interval. *** *P* < 0.001.

**Fig. S2 Comparison of *Q*_10_ values for different permafrost types.** Error bars in the bar charts represent standard errors (continuous permafrost, *n* = 8; discontinuous permafrost, *n* = 16; isolated permafrost, *n* = 10; sporadic permafrost, *n* = 6). The overall dataset includes 30 *Q_1_*_0_ values obtained from this study and 10 additional *Q*_10_ values synthesized from published literature across Arctic permafrost regions (Supplementary References List 1). The Kruskal-Wallis rank sum test, followed by Dunn’s post hoc test, was conducted to determine significant differences among permafrost types. Different lowercase letters indicated significant differences (*P* < 0.05) across various permafrost types. The permafrost types were derived from the Circum-Arctic map of permafrost [48], and classified as follows: continuous permafrost (covering 90-100% of the landscape), discontinuous permafrost (50-90%), sporadic permafrost (10-50%), and isolated patches (0.5-10%) [70].

**Fig. S3** **Comparison of average peak growing-season (July-August) enhanced vegetation index (EVI) across different permafrost types during 2000-2022.** Error bars in the bar charts represent standard errors (continuous permafrost, *n* = 8; discontinuous permafrost, *n* = 16; isolated permafrost, *n* = 10; sporadic permafrost, *n* = 6). The Kruskal-Wallis rank sum test, followed by Dunn’s post hoc test, was conducted to determine significant differences among permafrost types. Different lowercase letters indicated significant differences (*P* < 0.05) across various permafrost types. The permafrost type was derived from the Circum-Arctic map of permafrost [48]. Permafrost types were classified as follows: continuous permafrost (covering 90-100% of the landscape), discontinuous permafrost (50-90%), sporadic permafrost (10-50%), and isolated patches (0.5-10%) [70]. Monthly EVI data were obtained from the MOD13A3.061 dataset ([https://appeears.earthdatacloud.nasa.gov/) by](https://appeears.earthdatacloud.nasa.gov/)%20by) converting the 16-day composite data based on the maximum value composite method [71].

**Fig. S4 Ecological predictors of soil *Q*_10_ variation during short-term (three weeks) incubation.** (a) Structural equation model (SEM) illustrates the direct and indirect effects of climate, soil properties, substrate quantity, mineral protection, and microbial properties on soil *Q*_10_ variation. (b) Direct effects of mineral protection and microbial properties, and indirect effects of climate, soil properties and substrate quantity. Arrows represent causal relationships, with red and blue arrows indicating positive and negative effects, respectively. The numbers next to the arrows represent the strength of the effects. Climate is represented by aridity (1 - aridity index), with higher values indicating greater aridity. Soil properties were represented by pH and clay + silt content, and substrate quantity by soil organic carbon (SOC). Mineral protection is shown by the proportion of Ca-bound organic carbon (OC-Ca). Microbial properties include the bacteria positive/negative taxa, fungi positive/negative taxa, Shannon index of bacteria and fungi, community-weighted *rrn* copy number, the ratio of symbiotic to saprophytic fungi (Sym/Sap), and the abundances of C-degrading functional genes (*apu*, *sga*, *xylA*, *abfA*, *manB*, *cex*, *chiA*, *glx*, and *lig*). Bacteria positive/negative taxa were identified by aggregating dominant phyla that were positively/negatively correlated with *Q*_10_ (following the method in ref. [72]). Fungi positive/negative taxa were defined using a method analogous to that employed for bacteria. * *P* < 0.05, ** *P* < 0.01, and *** *P* < 0.001.

**Fig. S5** **Ecological predictors of soil *Q*_10-q_ variation.** (a) Structural equation model (SEM) illustrates the direct and indirect effects of climate, soil properties, substrate quantity, mineral protection, and microbial properties on soil *Q*_10-q_ variation at cumulative respired soil C in the range of 0.2–0.4%. (b) Standardized direct effects of mineral protection and microbial properties, as well as indirect effects of climate and soil properties on *Q*_10-q_ variation at cumulative respired soil C in the range of 0.2–0.4%. (c) SEM illustrates the direct and indirect effects of the above factors on soil *Q*_10-q_ variation at cumulative respired soil C in the range of 0.6–0.8%. (d) Standardized direct effects of mineral protection and microbial properties, as well as indirect effects of climate and soil properties on *Q*_10-q_ variation at 0.6–0.8% cumulative respired soil C. *Q*_10-q_s were determined by dividing the time taken to respire a given fraction of soil organic carbon (SOC) at 5°C by that at 15°C [20]. Arrows represent causal relationships, with red and blue arrows indicating positive and negative effects, respectively. The numbers next to the arrows represent the strength of the effects. Climate is represented by aridity (1 - aridity index), with higher values indicating greater aridity. Soil properties are represented by pH and clay + silt content, and substrate quantity by SOC. Mineral protection is shown by the proportion of Ca-bound organic carbon (OC-Ca). Microbial properties include the bacteria positive/negative taxa, fungi positive/negative taxa, Shannon index of bacteria and fungi, community-weighted *rrn* copy number, the ratio of symbiotic to saprophytic fungi (Sym/Sap), and the abundances of C-degrading functional genes (*apu*, *sga*, *xylA*, *abfA*, *manB*, *cex*, *chiA*, *glx*, and *lig*). Bacteria positive/negative taxa were identified by aggregating dominant phyla that were positively/negatively correlated with *Q*_10-qS_ (following the method in ref. [72]). Fungi positive/negative taxa were defined using the method analogous to that employed for bacteria. * *P* < 0.05, ** *P* < 0.01, and *** *P* < 0.001.

**Fig. S6** **Correlations between soil *Q*_10_ and dominant microbial phyla.** (a) Spearman correlations between soil *Q*_10_ and various microbial phyla. (b-e) Relationships of soil *Q*_10_ with the relative abundance of Proteobacteria (b), Actinobacteriota (c), Mortierellomycota (d), and Ascomycota (e). Bacteria positive/negative taxa were identified by aggregating dominant phyla that were positively/negatively correlated with *Q*_10._ Fungi positive/negative taxa were defined using a method analogous to that employed for bacteria (following the method in ref. [72]). Scatter plots show the taxa with the highest R² within each group (bacteria positive/negative taxa and fungi positive/negative taxa). TP and NE represent permafrost regions of the Tibetan Plateau and Northeast China, respectively. The solid line denotes the significant linear/nonlinear regression, and the grey shaded area is 95% confidence interval. * *P* < 0.05, ** *P* < 0.01, and *** *P* < 0.001.

**Fig. S7** **Soil microbial cross-kingdom co-occurrence networks** **of bacterial and fungal taxa in permafrost regions of the Tibetan Plateau (a) and Northeast China (b).** In the network, nodes represent ASVs, and links denote correlations between nodes. The size of each node is proportional to the number of correlations. Blue and yellow links denote positive and negative interactions with strong (*r* > 0.8 or *r* < -0.8) and significant (*P* < 0.001) correlations, respectively. Nodes are colored according to their phylum taxonomy. For details on the construction of microbial networks, refer to Supplementary Note 4.

**Fig. S8 Relationships between microbial life history strategies and SOM chemical components in permafrost regions of the Tibetan Plateau and Northeast China.** (a, b) Associations of *rrn* copy number with O-alkyl C (a) and Aromatic C (b). (c, d) Relationships of Sym/Sap with O-alkyl C (c) and Aromatic C (d). Sym/Sap, the ratio of symbiotic fungi to saprophytic fungi. SOM, soil organic matter. TP and NE represent permafrost regions of the Tibetan Plateau and Northeast China, respectively. The solid line denotes the significant linear regression, and the grey shaded area is 95% confidence interval. * *P* < 0.05, ** *P* < 0.01, and *** *P* < 0.001.

**Fig. S9 Relationship between OC-Ca content and aridity**. OC-Ca, the proportion of Ca-bound organic C. Aridity was expressed as1-aridity index, where higher values indicate greater aridity. TP and NE represent permafrost regions of the Tibetan Plateau and Northeast China, respectively. The solid line denotes the significant linear regression, and the grey shaded area is 95% confidence interval. ** *P* < 0.01.

**Fig. S10 Relationships of *Q*_10_ values, microbial life history strategies, and OC-Ca content with soil pH in the permafrost regions of the Tibetan Plateau and Northeast China.** (a) Relationship between *Q*_10_ values and soil pH. (b) Association of *rrn* copy number with soil pH. (c) Association of Sym/Sap with pH. (d) Relationship between OC-Ca content and soil pH. OC-Ca, the proportion of Ca-bound organic C. Sym/Sap, the ratio of symbiotic fungi to saprophytic fungi.TP and NE represent permafrost regions of the Tibetan Plateau and Northeast China, respectively. The solid line denotes the significant linear regression, and the grey shaded area is 95% confidence interval. *** *P* < 0.001.

**Fig. S11** **Density distributions of climate variables at sampling sites selected from permafrost regions of the Tibetan Plateau (24 sites) and Northeast China (30 sites).** (a, b) MAT, mean annual temperature. (c, d) MAP, mean annual precipitation. TP and NE represent permafrost regions of the Tibetan Plateau and Northeast China, respectively. Data were obtained from the National Tibetan Plateau/Third Pole Environment Data Center (https://data.tpdc.ac.cn/home). MAT (<https://data.tpdc.ac.cn/zh-hans/data/71ab4677-b66c-4fd1-a004-b2a541c4d5bf>), MAP (https://data.tpdc.ac.cn/zh-hans/data/faae7605-a0f2-4d18-b28f-5cee413766a2).

**Fig. S12** **Comparison of mean monthly temperatures during the peak growing season (July–August) from 1970 to 2022 in the high-latitude permafrost regions of the Tibetan Plateau and Northeast China**. Boxes represent the interquartile range (25th to 75th percentiles), with horizontal lines indicating the mean values. Whiskers extend to 1.5 times the interquartile range. TP and NE refer to permafrost regions of the Tibetan Plateau and Northeast China, respectively. The Wilcoxon rank-sum test was used to assess significant differences between TP and NE. *** *P* < 0.001. Data were obtained from the National Tibetan Plateau/Third Pole Environment Data Center (<https://data.tpdc.ac.cn/zh-hans/data/71ab4677-b66c-4fd1-a004-b2a541c4d5bf>).

**Fig. S13** **Variations in CO_2_ release rate and cumulative respired soil C during the incubation period at 5°C and 15°C.** (a) CO_2_ release rate. (b) Cumulative respired soil carbon (C). Cumulative respired soil C was converted from the cumulative C mineralization, which was calculated by multiplying the mean CO_2_ concentrations per unit of organic C between two consecutive measurements by the corresponding time interval and summing the values over the entire incubation period [73]. Error bars are standard error (*n* =15). TP and NE represent permafrost regions of the Tibetan Plateau and Northeast China, respectively.

**Fig. S14** **SOM chemical structures in the permafrost regions of the Tibetan Plateau and Northeast China based on solid-state ^13^C CP/MAS NMR spectra.** The chemical shifts are categorized into the following functional groups: 0-50 ppm (Alkyl C), 50-110 ppm (O-alkyl C), 110-165 ppm (Aromatic C), 165-220 ppm (Carboxylic C) [25]. TP and NE represent permafrost regions of the Tibetan Plateau and Northeast China, respectively. The spectra shown represents one representative sample from each region.

**Fig. S15** **A priori structural equation model (SEM) describing the effects of multiple ecological predictors on the *Q*_10_ of soil organic matter (SOM) decomposition.** The primary pathways in the model include (1) climate affects *Q*_10_ indirectly through soil properties, mineral protection, substrate quantity and microbial properties; (2) soil properties affect *Q*_10_ either directly or indirectly by altering substrate quantity, mineral protection, and microbial properties; (3) substrate quantity influences *Q*_10_ either directly or indirectly by affecting mineral protection, and microbial properties; (4) soil microorganisms as ultimate actors SOM decomposition directly affect *Q*_10_; and (5) minerals affect *Q*_10_ either by directly stabilizing SOM or by inhibiting microbial decomposition. Climate variable is represented by aridity (1-aridity index), with higher values indicating greater aridity. Soil properties are represented by pH and clay+silt content. Substrate quantity comprises soil organic carbon (SOC). Mineral protection is indicated by the proportion of Ca-bound organic C (OC-Ca). Microbial compositions were represented by aggregating dominant phyla positively correlated with *Q*_10_ to define “bacteria positive taxa”, while those negatively correlated with *Q*_10_ were grouped as “bacteria negative taxa” (following the method in ref. [72]). A similar approach was applied to fungi. The microbial properties also included Shannon index of bacteria and fungi, community-weighted *rrn* copy number, the ratio of symbiotic to saprophytic fungi (Sym/Sap), and the abundance of C-degrading functional genes (*apu*, *sga*, *xylA*, *abfA*, *manB*, *cex*, *chiA*, *glx*, and *lig*).

**Fig. S16** **Comparison of active layer thickness across different permafrost types.** The dataset includes sampling sites from the Tibetan Plateau and Northeast China, as well as Arctic sites compiled from published sources. Error bars in the bar chart represent standard errors (continuous permafrost, *n* = 5; discontinuous permafrost, *n* = 14; isolated permafrost, *n* = 7; sporadic permafrost, *n* = 6). The Kruskal–Wallis rank sum test followed by Dunn’s post hoc test was used to assess differences among permafrost types. Different lowercase letters indicate significant differences (*P* < 0.05) among permafrost types. Active layer data for the Arctic permafrost regions were obtained from the Circumpolar Active Layer Monitoring (CALM) network (<https://www2.gwu.edu/~calm/data/data-links.htm>). The corresponding data for the Tibetan alpine permafrost regions were obtained from field measurements, whereas data for the discontinuous and isolated permafrost in Northeast China were sourced from ref. [10]. The permafrost types were derived from the Circum-Arctic map of permafrost [48], and classified as the following standard: continuous permafrost (covering 90-100% of the landscape), discontinuous permafrost (50-90%), sporadic permafrost (10-50%), and isolated patches (0.5-10%) [70].

**Fig. S17** **Linear discriminant analysis effect size (LEfSe) for bacterial (a) and fungal taxa (b) between the permafrost regions of the Tibetan Plateau and Northeast China.** Cladograms illustrate the top 40 significantly enriched bacterial and fungal taxa from phyla to genera levels. Significant differences are indicated by LDA score > 4 and *P* < 0.01. TP and NE represent permafrost regions of the Tibetan Plateau and Northeast China, respectively. p_, phylum; c_, class; o_, order; f_, family; g_, genus.

**Table S1. Summary of site characteristics across permafrost regions of the Tibetan Plateau and Northeast China.**

| **Sites** | | **Altitude**  **(m)** | | **MAT**  **(°C)** | **MAP**  **(mm)** | **Vegetation type** | **Permafrost type** | | | **Soil type** |
| --- | --- | --- | --- | --- | --- | --- | --- | --- | --- | --- |
| 1 | 4597 | | -3.0 | | 449.3 | Swamp meadow | | Sporadic | | Cambisols |
| 2 | 4479 | | -1.3 | | 408.7 | Swamp meadow | | Sporadic | | Cambisols |
| 3 | 4459 | | 1.8 | | 352.4 | Alpine meadow | | Sporadic | | Cambisols |
| 4 | 3774 | | -0.4 | | 279.7 | Alpine meadow | | Sporadic | | Cambisols |
| 5 | 4042 | | -1.0 | | 279.7 | Swamp meadow | | Sporadic | | Calcisols |
| 6 | 4761 | | -3.6 | | 373.7 | Alpine meadow | | Discontinuous | | Calcisols |
| 7 | 4828 | | -2.6 | | 468.8 | Alpine meadow | | Discontinuous | | Cambisols |
| 8 | 5078 | | -1.8 | | 479.9 | Alpine meadow | | Discontinuous | | Cambisols |
| 9 | 4584 | | -3.5 | | 350.2 | Alpine meadow | | Sporadic | | Calcisols |
| 10 | 4884 | | -3.7 | | 350.2 | Alpine meadow | | Discontinuous | | Cambisols |
| 11 | 4913 | | -3.7 | | 350.2 | Alpine meadow | | Discontinuous | | Cambisols |
| 12 | 4580 | | -3.9 | | 320.5 | Alpine meadow | | Discontinuous | | Calcisols |
| 13 | 4640 | | -4.5 | | 272.5 | Alpine steppe | | Discontinuous | | Cryosols |
| 14 | 4523 | | -0.2 | | 244.6 | Alpine steppe | | Discontinuous | | Cryosols |
| 15 | 4668 | | 1.3 | | 244.6 | Alpine steppe | | Discontinuous | | Cambisols |
| 16 | 575 | | -3.2 | | 461.8 | Deciduous coniferous forest | | Discontinuous | | Cambisols |
| 17 | 301 | | -3.5 | | 427.8 | Deciduous coniferous forest | | Isolated | | Cambisols |
| 18 | 347 | | -2.1 | | 444.2 | Deciduous coniferous forest | | Discontinuous | | Cambisols |
| 19 | 463 | | -2.0 | | 466.5 | Mixed conifer-broadleaf forest | | Discontinuous | | Cambisols |
| 20 | 460 | | -1.1 | | 471.9 | Mixed conifer-broadleaf forest | | Isolated | | Cambisols |
| 21 | 504 | | -0.3 | | 478.3 | Mixed deciduous broadleaf forest | | | Isolated | Cambisols |
| 22 | 652 | | -2.2 | | 468.1 | Mixed conifer-broadleaf forest | | Discontinuous | | Cambisols |
| 23 | 781 | | -3.7 | | 458.6 | Mixed conifer-broadleaf forest | | Discontinuous | | Cambisols |
| 24 | 520 | | -1.6 | | 325.7 | Typical steppe | | Isolated | | Chernozerns |
| 25 | 742 | | 0.5 | | 265.9 | Typical steppe | | Isolated | | Kastanozerns |
| 26 | 670 | | -1.7 | | 333.2 | Typical steppe | | Isolated | | Chernozerns |
| 27 | 736 | | -3.0 | | 433.2 | Deciduous coniferous forest | | Isolated | | Phaeozerns |
| 28 | 895 | | -0.4 | | 430.4 | Mixed conifer-broadleaf forest | | Isolated | | Cambisols |
| 29 | 389 | | 0.8 | | 566.4 | Mixed conifer-broadleaf forest | | Isolated | | Cambisols |
| 30 | 572 | | -0.4 | | 521.1 | Deciduous coniferous forest | | Isolated | | Cambisols |

*Notes*: Sites 1-15 are located in permafrost region of the Tibetan Plateau, while sites 16-30 are situated in the permafrost region of Northeast China. MAT, mean annual temperature; MAP, mean annual precipitation. Permafrost types were derived from the Circum-Arctic permafrost map [48], and classified into the following categories: discontinuous permafrost (covering 50-90% of the landscape), sporadic permafrost (10-50%), and isolated patches (0.5-10%) [70].

**Table S2. Results of principal component analysis of soil properties and microbial properties.**

| **Factors** | **Variables** | **PC1** |
| --- | --- | --- |
| **Soil properties** | pH | 0.92** |
|  | Clay+silt | -0.92** |
| Cumulative (%) |  | **85.4** |
| **Microbial properties** | Bacteria positive taxa | 0.67** |
|  | Bacteria negative taxa | -0.65** |
|  | Bacterial Shannon | 0.20* |
|  | *rrn* copy number | -0.39* |
|  | Fungi positive taxa | 0.84** |
|  | Fungi negative taxa | -0.53** |
|  | Fungi Shannon | 0.54** |
|  | Sym/Sap | 0.51** |
|  | *apu* | 0.85** |
|  | *sga* | 0.92** |
|  | *xylA* | 0.93** |
|  | *abfA* | 0.92** |
|  | *manB* | 0.96** |
|  | *cex* | 0.85** |
|  | *chiA* | 0.95** |
|  | *glx* | 0.93** |
|  | *lig* | 0.96** |
| Cumulative (%) |  | **60.1** |

*Notes*: The first component of PCA analysis illustrates the correlation coefficients of soil and microbial properties, as well as the proportion of variance explained by the principal components. * and ** means *P* < 0.05 and *P* < 0.01, respectively. Microbial compositions were represented by aggregating dominant phyla positively correlated with *Q*_10_ to define “bacteria positive taxa”, while those negatively correlated with *Q*_10_ were grouped as “bacteria negative taxa” (following the method in ref. [72]). A similar approach was applied to fungi; Sym/Sap, the ratio of symbiotroph to saprotroph fungi.

**Table S3. Analyses of variance inflation factors (VIF).** The VIF values were shown for the hypothetical drivers of *Q*_10_. The range of VIF < 10 indicates no potential multicollinearity issues [72].

| **Factors** | **VIF** |
| --- | --- |
| Climate | 2.34 |
| Soil properties | 9.41 |
| Substrate quantity | 4.25 |
| Mineral protection | 6.37 |
| Microbial properties | 4.48 |

*Notes*: Climate is represented by aridity (1-aridity index), with higher values indicating greater aridity. Soil and microbial properties are captured by the extracted PC1 axis, respectively. Soil properties include pH and clay + silt content. Substrate quantity comprises soil organic carbon (SOC). Mineral protection properties include the proportion of Ca-bound organic C (OC-Ca). Microbial properties include bacteria positive/negative taxa, fungi positive/negative taxa, Shannon index of bacteria and fungi, community-weighted *rrn* copy number, the ratio of symbiotic to saprophytic fungi (Sym/Sap), and the abundance of C-degrading functional genes (*apu*, *sga*, *xylA*, *abfA*, *manB*, *cex*, *chiA*, *glx*, and *lig*). Bacteria positive/negative taxa were identified by aggregating dominant phyla that were positively/negatively correlated with *Q*_10._ Fungi positive/negative taxa were defined using a method analogous to that employed for bacteria (following the method in ref. [72]).

**Table S4. List of individual soil taxa (ASVs) positively or negatively correlated with soil *Q*_10_ across high-latitude and high-altitude permafrost regions.**

Table S4 is available online as a separate .xlsx file included in the Supplementary Data for this article.

**Table S5. The name and annotation of genes related to carbon (C) cycling detected by high-throughput based chip [47].**

| **Gene name** | **Classification** | **Gene category** | **Function** |
| --- | --- | --- | --- |
| *apu* | C degradation | Starch | Amylopullulanase |
| *sga* | C degradation | Starch | Glucoamylase |
| *xylA* | C degradation | Hemicellulose | Xylose isomerase |
| *abfA* | C degradation | Hemicellulose | α-L-arabinofuranosidase |
| *manB* | C degradation | Hemicellulose | Hemicellulose |
| *cdh* | C degradation | Cellulose | Cellobiose dehydrogenase |
| *cex* | C degradation | Cellulose | Exoglucanase |
| *chiA* | C degradation | Chitin | Endochitinase |
| *pgu* | C degradation | Pectin | Pectinase/polygalacturonase |
| *glx* | C degradation | Lignin | Glyoxal oxidase |
| *lig* | C degradation | Lignin | Lignin peroxidase |
| *mnp* | C degradation | Lignin | Manganese peroxidase |

**Reference list 1. Literatures used to synthesize *Q*_10_ data in Arctic permafrost region.**

1.	Gentsch N, Wild B, Mikutta R *et al.* Temperature response of permafrost soil carbon is attenuated by mineral protection. *Glob Change Biol* 2018; **24**: 3401-15.

2.	Hobbie SE, Miley TA, Weiss MS. Carbon and nitrogen cycling in soils from acidic and nonacidic tundra with different glacial histories in Northern Alaska. *Ecosystems* 2002; **5**: 0761-74.

3.	Lavoie M, Mack M, Schuur E. Effects of elevated nitrogen and temperature on carbon and nitrogen dynamics in Alaskan arctic and boreal soils. *J Geophys Res: Biogeosci* 2011; **116**.

4.	Nadelhoffer K, Giblin A, Shaver G *et al.* Effects of temperature and substrate quality on element mineralization in six arctic soils. *Ecology* 1991; **72**: 242-53.

5.	Ren S, Ding J, Yan Z *et al.* Higher temperature sensitivity of soil C release to atmosphere from northern permafrost soils as indicated by a meta‐analysis. *Glob Biogeochem Cycles* 2020; **34**: e2020GB006688.

6.	Rodionow A, Flessa H, Kazansky O *et al.* Organic matter composition and potential trace gas production of permafrost soils in the forest tundra in northern Siberia. *Geoderma* 2006; **135**: 49-62.

**Reference list 2. Literatures cited in the supplementary file.**

1.	Zhao L, Sheng Y. Permafrost and its changes on the Qinghai-Tibetan Plateau. Beijing: Science Press, 2019.

2.	Li H, Wu Y, Liu S *et al.* Decipher soil organic carbon dynamics and driving forces across China using machine learning. *Glob Change Biol*. 2022; **28**: 3394-410.

3.	Ding J, Chen L, Zhang B *et al.* Linking temperature sensitivity of soil CO_2_ release to substrate, environmental, and microbial properties across alpine ecosystems. *Glob Biogeochem Cycles*. 2016; **30**: 1310-23.

4.	Chen L, Liu L, Qin S *et al.* Regulation of priming effect by soil organic matter stability over a broad geographic scale. *Nat Commun*. 2019; **10**: 5112.

5.	IUSS Working Group WRB. 2015. World reference base for soil resources 2014, update 2015. International soil classification system for naming soils and creating legends for soil maps. World Soil Resources Reports No. 106. Rome, Italy: FAO.

6.	Ding J, Chen L, Ji C *et al.* Decadal soil carbon accumulation across Tibetan permafrost regions. *Nat Geosci*. 2017; **10**: 420-24.

7.	Jin H, Li S, Cheng G *et al.* Permafrost and climatic change in China. *Global Planet Change*. 2000; **26**: 387-404.

8.	Zhu X, Wu T, Ni J *et al.* Increased extreme warming events and the differences in the observed hydrothermal responses of the active layer to these events in China’s permafrost regions. *Clim Dyn*. 2022; **59**: 785-804.

9.	Wang H, Jin H, Che T *et al.* Influences of snow cover on the thermal regimes of Xing'an permafrost in Northeast China in 1960s–2010s. *Permafr Periglac Process*. 2024; **35**: 188-201.

10.	Huang S, Ding Q, Chen K *et al.* Changes in near-surface permafrost temperature and active layer thickness in Northeast China in 1961–2020 based on GIPL model. *Cold Reg Sci Technol*. 2023; **206**: 103709.

11.	Mao C, Kou D, Chen L *et al.* Permafrost nitrogen status and its determinants on the Tibetan Plateau. *Glob Change Biol*. 2020; **26**: 5290-302.

12.	Yang Y, Jia B, He N, et al. Soil carbon storage and its spatial distribution in the permafrost region of Northeast China (in Chinese). *Sci Sin Vitae*, 2025; **55**:1716-30. https://doi. 10.1360/SSV-2025-0026.

13.	Li J, Yan D, Pendall E *et al.* Depth dependence of soil carbon temperature sensitivity across Tibetan permafrost regions. *Soil Biol Biochem*. 2018; **126**: 82-90.

14.	Qin S, Kou D, Mao C *et al.* Temperature sensitivity of permafrost carbon release mediated by mineral and microbial properties. *Sci Adv*. 2021; **7**: eabe3596.

15.	Dutta K, Schuur E, Neff J *et al.* Potential carbon release from permafrost soils of Northeastern Siberia. *Glob Change Biol*. 2006; **12**: 2336-51.

16.	Gentsch N, Wild B, Mikutta R *et al.* Temperature response of permafrost soil carbon is attenuated by mineral protection. *Glob Change Biol*. 2018; **24**: 3401-15.

17.	Fang K, Chen L, Qin S *et al.* Mineral and climatic controls over soil organic matter stability across the Tibetan alpine permafrost region. *Glob Biogeochem Cycles*. 2021; **35**: e2021GB007118.

18.	Zhang S, Wang M, Xiao L *et al.* Reconciling carbon quality with availability predicts temperature sensitivity of global soil carbon mineralization. *Proc Natl Acad Sci USA*. 2024; **121**: e2313842121.

19.	Wen S, Hu A, Jiang S *et al.* Temperature sensitivity of organic carbon decomposition in lake sediments is mediated by chemodiversity. *Glob Change Biol*. 2024; **30**: e17158.

20.	Conant RT, Drijber RA, Haddix ML *et al.* Sensitivity of organic matter decomposition to warming varies with its quality. *Glob Change Biol*. 2008; **14**: 868-77.

21.	Rey A, Jarvis P. Modelling the effect of temperature on carbon mineralization rates across a network of European forest sites (FORCAST). *Glob Change Biol*. 2006; **12**: 1894-908.

22.	Zomer RJ, Xu J, Trabucco A. Version 3 of the global aridity index and potential evapotranspiration database. *Sci Data*. 2022; **9**: 409.

23.	Díaz-Martínez P, Maestre FT, Moreno-Jiménez E *et al.* Vulnerability of mineral-associated soil organic carbon to climate across global drylands. *Nat Clim Change*. 2024; **14**: 976-82.

24.	Hall SJ, Ye C, Weintraub SR *et al.* Molecular trade-offs in soil organic carbon composition at continental scale. *Nat Geosci*. 2020; **13**: 687-92.

25.	Simpson MJ, Simpson AJ. The chemical ecology of soil organic matter molecular constituents. *J Chem Ecol*. 2012; **38**: 768-84.

26.	Wagai R, Kishimoto‐Mo AW, Yonemura S *et al.* Linking temperature sensitivity of soil organic matter decomposition to its molecular structure, accessibility, and microbial physiology. *Glob Change Biol*. 2013; **19**: 1114-25.

27.	Lavallee JM, Soong JL, Cotrufo MF. Conceptualizing soil organic matter into particulate and mineral‐associated forms to address global change in the 21st century. *Glob Change Biol*. 2020; **26**: 261-73.

28.	Patzner MS, Mueller CW, Malusova M *et al.* Iron mineral dissolution releases iron and associated organic carbon during permafrost thaw. *Nat Commun*. 2020; **11**: 6329.

29.	Xu J, Yuan K. Dissolution and fractionation of calcium-bound and iron-and aluminumbound humus in soils. *Pedosphere.* 1993; **3**:75-80.

30.	Finkel OM, Salas-González I, Castrillo G *et al.* A single bacterial genus maintains root growth in a complex microbiome. *Nature*. 2020; **587**: 103-08.

31.	White TJ, Bruns T, Lee S *et al.* Amplification and direct sequencing of fungal ribosomal RNA genes for phylogenetics. *PCR protocols: a guide to methods and applications*. 1990; **18**: 315-22.

32.	Andrews S. FastQC: a quality control tool for high throughput sequence data http://www. bioinformatics. babraham. ac. uk/projects/fastqc. *Babraham Bioinformatics*. 2010.

33.	Edgar RC. Search and clustering orders of magnitude faster than BLAST. *Bioinformatics*. 2010; **26**: 2460-61.

34.	Rognes T, Flouri T, Nichols B *et al.* VSEARCH: a versatile open source tool for metagenomics. *PeerJ*. 2016; **4**: e2584.

35.	Edgar RC. UNOISE2: improved error-correction for Illumina 16S and ITS amplicon sequencing. *bioRxiv*. 2016: 081257. <https://doi.org/10.1101/081257>.

36.	Oksanen J, Blanchet FG, Kindt R *et al.* Community ecology package. *R package version*. 2013; **2**: 321-26.

37.	R Core Team R: A Language and Environment for Statistical Computing. Vienna, Austria: R Foundation for Statistical Computing; 2022.

38.	Liu C, Cui Y, Li X *et al.* microeco: an R package for data mining in microbial community ecology. *FEMS Microbiol Ecol*. 2021; **97**: fiaa255.

39.	Segata N, Izard J, Waldron L *et al.* Metagenomic biomarker discovery and explanation. *Genome Biol*. 2011; **12**: 1-18.

40.	Klappenbach JA, Dunbar JM, Schmidt TM. rRNA operon copy number reflects ecological strategies of bacteria. *Appl Environ Microbiol*. 2000; **66**: 1328-33.

41.	Lee ZMP, Bussema III C, Schmidt TM. rrn DB: documenting the number of rRNA and tRNA genes in bacteria and archaea. *Nucleic Acids Res*. 2009; **37**: D489-D93.

42.	Stoddard SF, Smith BJ, Hein R *et al.* rrn DB: improved tools for interpreting rRNA gene abundance in bacteria and archaea and a new foundation for future development. *Nucleic Acids Res*. 2015; **43**: D593-D98.

43.	Nemergut DR, Knelman JE, Ferrenberg S *et al.* Decreases in average bacterial community rRNA operon copy number during succession. *ISME J*. 2016; **10**: 1147-56.

44.	Nguyen NH, Song Z, Bates ST *et al.* FUNGuild: an open annotation tool for parsing fungal community datasets by ecological guild. *Fungal Ecol*. 2016; **20**: 241-48.

45.	Li H, Yang S, Semenov MV *et al.* Temperature sensitivity of SOM decomposition is linked with a K‐selected microbial community. *Glob Change Biol*. 2021; **27**: 2763-79.

46.	Lindahl BD, Ihrmark K, Boberg J *et al.* Spatial separation of litter decomposition and mycorrhizal nitrogen uptake in a boreal forest. *New Phytol*. 2007; **173**: 611-20.

47.	Dai Z, Guo X, Lin J *et al.* Metallic micronutrients are associated with the structure and function of the soil microbiome. *Nat Commun*. 2023; **14**: 8456.

48.	Brown J, Ferrians O, Heginbottom J *et al.* Circum-Arctic map of permafrost and ground-ice conditions, version 2. National Snow and Ice Data Center. Colorado USA Boulder; 2002. [https://doi.org/10.7265/skbgkf16](about:blank).

49.	Nottingham AT, Scott JJ, Saltonstall K *et al.* Microbial diversity declines in warmed tropical soil and respiration rise exceed predictions as communities adapt. *Nat Microbiol*. 2022; **7**: 1650-60.

50.	Biskaborn BK, Smith SL, Noetzli J *et al.* Permafrost is warming at a global scale. *Nat Commun*. 2019; **10**: 264.

51.	Peng X, Zhang T, Frauenfeld OW *et al.* Northern Hemisphere greening in association with warming permafrost. *J Geophys Res: Biogeosci*. 2020; **125**: e2019JG005086.

52.	Hu G, Zhao L, Wu T *et al.* Spatiotemporal variations and regional differences in air temperature in the permafrost regions in the Northern Hemisphere during 1980–2018. *Sci Total Environ*. 2021; **791**: 148358.

53.	Bell CW, Acosta-Martinez V, McIntyre NE *et al.* Linking microbial community structure and function to seasonal differences in soil moisture and temperature in a Chihuahuan desert grassland. *Microb Ecol*. 2009; **58**: 827-42.

54.	He M, Fang K, Chen L *et al.* Depth‐dependent drivers of soil microbial necromass carbon across Tibetan alpine grasslands. *Glob Change Biol*. 2022; **28**: 936-49.

55.	Chen L, Yang G, Bai Y *et al.* Permafrost carbon cycle and its dynamics on the Tibetan Plateau. *Sci China Life Sci*. 2024; **67**: 1833-1848.

56.	Jin X, Jin H, Iwahana G *et al.* Impacts of climate-induced permafrost degradation on vegetation: A review. *Adv Clim Change Res*. 2021; **12**: 29-47.

57.	Gutiérrez-Girón A, Diaz-Pines E, Rubio A *et al.* Both altitude and vegetation affect temperature sensitivity of soil organic matter decomposition in Mediterranean high mountain soils. *Geoderma*. 2015; **237**: 1-8.

58.	Yang Y, Fang J, Tang Y *et al.* Storage, patterns and controls of soil organic carbon in the Tibetan grasslands. *Glob Change Biol*. 2008; **14**: 1592-99.

59.	Yang S, Wu H, Wang Z *et al.* Linkages between the temperature sensitivity of soil respiration and microbial life strategy are dependent on sampling season. *Soil Biol Biochem*. 2022; **172**: 108758.

60.	Rowley MC, Grand S, Verrecchia ÉP. Calcium-mediated stabilisation of soil organic carbon. *Biogeochemistry*. 2018; **137**: 27-49.

61.	Sun X, Zhang L, Pei J *et al.* Regulatory relationship between quality variation and environment of Cistanche deserticola in three ecotypes based on soil microbiome analysis. *Sci Rep*. 2020; **10**: 6662.

62.	Zhan Y, Yan N, Miao X *et al.* Different responses of soil environmental factors, soil bacterial community, and root performance to reductive soil disinfestation and soil fumigant chloropicrin. *Front Microbiol*. 2021; **12**: 796191.

63.	Willms IM, Bolz SH, Yuan J *et al.* The ubiquitous soil verrucomicrobial clade ‘Candidatus Udaeobacter’shows preferences for acidic pH. *Environ Microbiol Rep*. 2021; **13**: 878-83.

64.	Kalam S, Basu A, Ahmad I *et al.* Recent understanding of soil acidobacteria and their ecological significance: a critical review. *Front Microbiol*. 2020; **11**: 580024.

65.	Velez-Martinez GA, Reyes-Ardila WL, Duque-Zapata JD *et al.* Soil bacteria and fungi communities are shaped by elevation influences in Colombian forest and páramo natural ecosystems. *Int Microbiol*. 2024; **27**: 377-91.

66.	Gorfer M, Mayer M, Berger H *et al.* High fungal diversity but low seasonal dynamics and ectomycorrhizal abundance in a Mountain Beech forest. *Microb Ecol*. 2021; **82**: 243-56.

67.	Friedman J, Alm EJ. Inferring correlation networks from genomic survey data. *PLoS Comput Biol.* 2012; **8**: e1002687.

68.	Wu H, Gao T, Hu A *et al.* Network complexity and stability of microbes enhanced by microplastic diversity. *Environ Sci Technol*. 2024; **58**: 4334-45.

69.	Zhou Y, Sun B, Xie B *et al.* Warming reshaped the microbial hierarchical interactions. *Glob Change Biol*. 2021; **27**: 6331-47.

70.	Obu J, Westermann S, Bartsch A *et al.* Northern Hemisphere permafrost map based on TTOP modelling for 2000–2016 at 1 km^2^ scale. *Earth Sci Rev*. 2019; **193**: 299-316.

71.	Huete A, Didan K, Miura T *et al.* Overview of the radiometric and biophysical performance of the MODIS vegetation indices. *Remote Sens Environ*. 2002; **83**: 195-213.

72.	Sáez-Sandino T, García-Palacios P, Maestre FT *et al.* The soil microbiome governs the response of microbial respiration to warming across the globe. *Nat Clim Change*. 2023; **13**: 1382-87.

73.	Xu M, Li X, Kuyper TW *et al.* High microbial diversity stabilizes the responses of soil organic carbon decomposition to warming in the subsoil on the Tibetan Plateau. *Glob Change Biol*. 2021; **27**: 2061-75.
